# Supplementary material for: Regional and country-level trends in cervical cancer screening coverage in sub-Saharan Africa: A systematic analysis of population-based surveys (2000–2020)
Source: PLoS Med. 2023 Jan 12;20(1):e1004143. doi: 10.1371/journal.pmed.1004143 (PMC9882915; doi:10.1371/journal.pmed.1004143)
Supplement: S1 Appendix — (PDF) [file pmed.1004143.s001.pdf]

S1 Appendix - Supporting Information

Table of Contents

Table A. GATHER checklist. 2

Table B. List of included surveys, their sample size, number screened for cervical cancer, survey questions, and sources. 4

Text A. Description of the methods and additional results for regional and national estimates of cervical cancer screening coverage time trends 11

Fig A. Country-level trends in lifetime and past three-year cervical cancer screening coverage among women aged 30-49 years between 2000-2020. 14

Fig B. Country-level trends of lifetime cervical cancer screening stratified by HIV status for women aged 25-49 years between 2000-2020. 15

Table C. Regional and country-level estimates of the percentage of women screened for cervical cancer in their lifetime and in the past three years in 2020 for countries with two or more surveys by five-year age groups between 30-49 years. 16

Fig C. Sensitivity analysis of country-level screening time trends with a fixed effect for national screening program. 17

Fig D. Sensitivity analysis of country-level screening time trends with a fixed effect for Gross National Income. 18

Fig E. Sensitivity analyses of regional-level trends in lifetime and past three years cervical cancer screening coverage among women aged 30-49 years. 19

Fig F. Posterior predictive checks comparing modelled estimates (red) to each empirical data point (blue) for the three regions. 22

Table D. In-sample comparisons of model fits with empirical survey observations. 23

Text B. Description of the methods and additional results for the WHO recommendations for frequency of re-screening. 24

Fig G. Conceptual framework to estimate re-screening rates and screening twice in a lifetime by age 45 outlining data inputs, data pre-processing, statistical analyses, and data post processing. 25

Fig H. Heat map of simulated first-time screening rates across 50 years for women 15-49 years. 27

Fig I. Re-screening model simulations comparison of simulated first-time screening rate and rate ratio estimates to modelled estimates. 30

Table E. Estimates of the rate ratio for rate of re-screening for cervical cancer as compared to rate of first-time screening with and without adjustment for telescoping bias. 31

Fig J. Robustness check of estimates for screening twice by the age of 45 years. 32

Fig K. Life table methods sensitivity analysis using various rate ratio values. 34

Text C. Description of the methods for cervical cancer treatment coverage. 35

Table F. Summary of main model assumptions and their justifications. 36

References 37

Table A. GATHER checklist.

Item #	Checklist item	Reported on page #
**Objectives and funding**		
**1**	Define the indicator(s), populations (including age, sex, and geographic entities), and time period(s) for which estimates were made.	Results: Survey Characteristics
**2**	List the funding sources for the work.	Funding
**Data Inputs**		
For all data inputs from multiple sources that are synthesized as part of the study:		
**3**	Describe how the data were identified and how the data were accessed.	Methods - *Data sources*
**4**	Specify the inclusion and exclusion criteria. Identify all ad-hoc exclusions.	Methods - *Data sources*
**5**	Provide information on all included data sources and their main characteristics. For each data source used, report reference information or contact name/institution, population represented, data collection method, year(s) of data collection, sex and age range, diagnostic criteria or measurement method, and sample size, as relevant.	Supplementary Materials - *Table B*
**6**	Identify and describe any categories of input data that have potentially important biases (e.g., based on characteristics listed in item 5).	NA
For data inputs that contribute to the analysis but were not synthesized as part of the study:		
**7**	Describe and give sources for any other data inputs.	Supplementary Materials - *Table B*
For all data inputs:		
**8**	Provide all data inputs in a file format from which data can be efficiently extracted (e.g., a spreadsheet rather than a PDF), including all relevant meta-data listed in item 5. For any data inputs that cannot be shared because of ethical or legal reasons, such as third-party ownership, provide a contact name or the name of the institution that retains the right to the data.	Supplementary Materials - *Table B*
**Data analysis**		
**9**	Provide a conceptual overview of the data analysis method. A diagram may be helpful.	Methods - *Fig 1*, *Text B*, *Fig G*
**10**	Provide a detailed description of all steps of the analysis, including mathematical formulae. This description should cover, as relevant, data cleaning, data pre-processing, data adjustments and weighting of data sources, and mathematical or statistical model(s).	Methods, Supplementary Materials
**11**	Describe how candidate models were evaluated and how the final model(s) were selected.	Methods – *Statistical analyses for the estimation of trends in screening coverage – paragraph 3*, *Text A*
**12**	Provide the results of an evaluation of model performance, if done, as well as the results of any relevant sensitivity analysis.	Results – *Regional and national estimates of cervical cancer screening coverage trends* – *Text A*, *Table D*, *Fig F*
**13**	Describe methods for calculating uncertainty of the estimates. State which sources of uncertainty were, and were not, accounted for in the uncertainty analysis.	Text A

**14**	State how analytic or statistical source code used to generate estimates can be accessed.	Data Sharing
**Results and Discussion**		
**15**	Provide published estimates in a file format from which data can be efficiently extracted.	Data Sharing
**16**	Report a quantitative measure of the uncertainty of the estimates (e.g., uncertainty intervals).	Results
**17**	Interpret results in light of existing evidence. If updating a previous set of estimates, describe the reasons for changes in estimates.	Discussion – *paragraph 1-5*
**18**	Discuss limitations of the estimates. Include a discussion of any modelling assumptions or data limitations that affect interpretation of the estimates.	Discussion – *paragraph 6-7*, *Table F*

Table B. List of included surveys, their sample size, number screened for cervical cancer, survey questions, and sources.

Country	Survey	Year	Sample size of women 25-49	Number screened 25-49[d]	Ever tested for cervical cancer	Timing of last cervical cancer test	Treatment as a result of last cervical cancer test	Survey Report	Source[e]
**Central Africa**									
**Republic of the Congo**	WHS	2003	637	210	Asked to those who've ever had a pelvic exam within the past 3 years: The last time you had the pelvic examination, did you have a Pap smear test? (By Pap smear test, I mean did a doctor or nurse use a swab or stick to wipe from inside your vagina, take a sample and send it to a laboratory?)	N/A	N/A	N/A	https://apps.who.int/healthinfo/systems/surveydata/index.php/catalog/103
**Eastern Africa**									
**Comoros**	WHS	2003	433	53	Asked to those who've ever had a pelvic exam within the past 3 years: The last time you had the pelvic examination, did you have a Pap smear test? (By Pap smear test, I mean did a doctor or nurse use a swab or stick to wipe from inside your vagina, take a sample and send it to a laboratory?)	N/A	N/A	N/A	https://apps.who.int/healthinfo/systems/surveydata/index.php/catalog/113
**Ethiopia**	WHS	2003	1321	15	Asked to those who've ever had a pelvic exam within the past 3 years: The last time you had the pelvic examination, did you have a Pap smear test? (By Pap smear test, I mean did a doctor or nurse use a swab or stick to wipe from inside your vagina, take a sample and send it to a laboratory?)	N/A	N/A	N/A	https://apps.who.int/healthinfo/systems/surveydata/index.php/catalog/37
**Ethiopia**	STEPS	2015	3276	116	Have you ever had a screening test for cervical cancer, using any of these methods described above (i.e., VIA, pap smear or HPV test)?	N/A	N/A	https://extranet.who.int/ncdsmicrodata/index.php/catalog/794/download/5522	https://extranet.who.int/ncdsmicrodata/index.php/catalog/794
**Ethiopia**	PHIA	2017-18	5882	343	Have you ever been tested for cervical cancer?	What month and year was your last test for cervical cancer?	N/A	https://phia.icap.columbia.edu/wp-content/uploads/2020/11/EPHIA_Report_280820_Web.pdf	https://phia-data.icap.columbia.edu/datasets?country_id=12
**Kenya**	WHS	2003	1373	83	Asked to those who've ever had a pelvic exam within the past 3 years: The last time you had the pelvic examination, did you have a Pap smear test? (By Pap smear test, I mean did a doctor or nurse use a swab or stick to wipe from inside your vagina, take a sample and send it to a laboratory?)	N/A	N/A	N/A	https://apps.who.int/healthinfo/systems/surveydata/index.php/catalog/80
**Kenya**	KAIS	2012	4255	416	Have you ever been screened by a doctor or other health professional for cervical cancer?	N/A	N/A	https://nacc.or.ke/wp-content/uploads/2015/10/KAIS-2012.pdf	http://catalog.ihsn.org/catalog/6697/study-description

**Kenya**	DHS	2014	9338	1454	Asked to those who've heard of cervical cancer: Have you ever had a test or exam to see if you had cervical cancer?	N/A	N/A	https://dhsprogram.com/pubs/pdf/FR308/FR308.pdf	https://dhsprogram.com/methodology/survey/survey-display-451.cfm
**Kenya**	STEPS	2015	1616	253	Have you ever had a screening test for cervical cancer, using any of these methods described above (i.e., VIA, pap smear or HPV test)?	N/A	N/A	https://extranet.who.int/ncdsmicrodata/index.php/catalog/247/download/2092	https://extranet.who.int/ncdsmicrodata/index.php/catalog/247
**Malawi**	WHS	2003	1366	44	Asked to those who've ever had a pelvic exam within the past 3 years: The last time you had the pelvic examination, did you have a Pap smear test? (By Pap smear test, I mean did a doctor or nurse use a swab or stick to wipe from inside your vagina, take a sample and send it to a laboratory?)	N/A	N/A	N/A	https://apps.who.int/healthinfo/systems/surveydata/index.php/catalog/85
**Malawi**	STEPS	2017	1561	279	Have you ever had a screening test for cervical cancer, using any of these methods described above (i.e., VIA, pap smear or HPV test)?	N/A	N/A	https://extranet.who.int/ncdsmicrodata/index.php/catalog/629/download/5770	https://extranet.who.int/ncdsmicrodata/index.php/catalog/629
**Malawi**	PHIA	2015-16	6062	926	Have you ever been tested for cervical cancer?	What month and year was your last test for cervical cancer?	Did you receive treatment after your last test for cervical cancer? Did you receive treatment on the same day or on a different day?	https://phia.icap.columbia.edu/wp-content/uploads/2020/02/MPHIA-Final-Report_web.pdf	https://phia-data.icap.columbia.edu/datasets?country_id=3
**Mauritius**	WHS	2003	1150	154	Asked to those who've ever had a pelvic exam within the past 3 years: The last time you had the pelvic examination, did you have a Pap smear test? (By Pap smear test, I mean did a doctor or nurse use a swab or stick to wipe from inside your vagina, take a sample and send it to a laboratory?)	N/A	N/A	N/A	https://apps.who.int/healthinfo/systems/surveydata/index.php/catalog/90
**Mozambique**[a]	STEPS	2015	697	24	Asked to those who've ever had a pelvic exam within the past 3 years: The last time you had the pelvic examination, did you have a Pap smear test? (By Pap smear test, I mean did a doctor or nurse use a swab or stick to wipe from inside your vagina, take a sample and send it to a laboratory?)	N/A	N/A	https://cdn.who.int/media/docs/default-source/ncds/ncd-surveillance/data-reporting/mozambique/relatorio_final_steps_2015_mozambique.pdf?sfvrsn=1907f08a_1&download=true	https://journals.lww.com/eurjcancerprev/Abstract/2019/07000/Cervical_cancer_screening_uptake_in_women_aged.13.aspx
**Rwanda**	PHIA	2018-19	8751	353	Have you ever been tested for cervical cancer?	What month and year was your last test	N/A	https://phia.icap.columbia.edu/wp-content/uploads/2020/	https://phia-data.icap.columbia.edu/datasets?country_id=11

						for cervical cancer?		11/RPHIA-Final-Report_Web.pdf	
**Uganda**	STEPS	2014	1387	143	Have you ever had a screening test for cervical cancer, using any of these methods described above (i.e., VIA, pap smear or HPV test)?	N/A	N/A	https://extranet.who.int/ncdsmicrodata/index.php/catalog/633/download/4536	https://extranet.who.int/ncdsmicrodata/index.php/catalog/633
**Tanzania**	PHIA	2016-17	9186	586	Have you ever been tested for cervical cancer?	What month and year was your last test for cervical cancer?	Did you receive treatment after your last test for cervical cancer? Did you receive treatment on the same day or on a different day?	https://phia.icap.columbia.edu/wp-content/uploads/2020/02/FINAL_THIS-2016-2017_Final-Report__06.21.19_for-web_TS.pdf	https://phia-data.icap.columbia.edu/datasets?country_id=10
**Zambia**	WHS	2003	1060	45	Asked to those who've ever had a pelvic exam within the past 3 years: The last time you had the pelvic examination, did you have a Pap smear test? (By Pap smear test, I mean did a doctor or nurse use a swab or stick to wipe from inside your vagina, take a sample and send it to a laboratory?)	N/A	N/A	N/A	https://apps.who.int/healthinfo/systems/surveydata/index.php/catalog/105
**Zambia**	PHIA	2016	6413	1066	Have you ever been tested for cervical cancer?	What month and year was your last test for cervical cancer?	Did you receive treatment after your last test for cervical cancer? Did you receive treatment on the same day or on a different day?	https://phia.icap.columbia.edu/wp-content/uploads/2020/02/ZAMPHIA-Final-Report__2.22.19.pdf	https://phia-data.icap.columbia.edu/datasets?country_id=5
**Zambia**	STEPS	2017	1398	280	Have you ever had a screening test for cervical cancer, using any of these methods described above (i.e., VIA, pap smear or HPV test)?	N/A	N/A	https://extranet.who.int/ncdsmicrodata/index.php/catalog/620/download/4457	https://extranet.who.int/ncdsmicrodata/index.php/catalog/620
**Southern Africa**									
**Botswana**	STEPS	2014	1507	705	Have you ever had a screening test for cervical cancer, using any of these methods described above (i.e., VIA, pap smear or HPV test)?	N/A	N/A	https://extranet.who.int/ncdsmicrodata/index.php/catalog/318/download/2484	https://extranet.who.int/ncdsmicrodata/index.php/catalog/318
**Eswatini**	WHS	2003	600	31	Asked to those who've ever had a pelvic exam within the past 3 years: The last time you had the pelvic examination, did you have a Pap smear test? (By Pap smear test, I mean did a doctor or nurse use a swab or stick to wipe from inside your vagina, take a sample and send it to a laboratory?)	N/A	N/A	N/A	https://apps.who.int/healthinfo/systems/surveydata/index.php/catalog/70

**Eswatini**	STEPS	2014	1104	207	Have you ever had a screening test for cervical cancer, using any of these methods described above (i.e., VIA, pap smear or HPV test)?	N/A	N/A	https://extranet.who.int/ncdsmicrodata/index.php/catalog/688/download/4938	https://extranet.who.int/ncdsmicrodata/index.php/catalog/688
**Lesotho**	DHS	2009	4226	335	Asked to those who've heard of a Pap smear: Have you ever had such an exam in your lifetime?	How long ago was the last exam performed?	N/A	https://dhsprogram.com/pubs/pdf/FR241/FR241.pdf	https://dhsprogram.com/methodology/survey/survey-display-317.cfm
**Lesotho**	DHS	2014	3779	528	Asked to those who've heard of a Pap smear: Have you ever had such an exam in your lifetime?	How long ago was the last exam performed?	N/A	https://dhsprogram.com/pubs/pdf/FR309/FR309.pdf	https://dhsprogram.com/methodology/survey/survey-display-462.cfm
**Namibia**	DHS	2000	3977	1275	Have you ever had a "Pap" smear to test for cervical cancer? PROBE: When a doctor or nurse takes a swab in your vagina and sends the slide to the laboratory for analysis?	N/A	N/A	https://dhsprogram.com/pubs/pdf/FR141/FR141.pdf	https://dhsprogram.com/methodology/survey/survey-display-205.cfm
**Namibia**	WHS	2003	1261	195	Asked to those who've ever had a pelvic exam within the past 3 years: The last time you had the pelvic examination, did you have a Pap smear test? (By Pap smear test, I mean did a doctor or nurse use a swab or stick to wipe from inside your vagina, take a sample and send it to a laboratory?)	N/A	N/A	N/A	https://apps.who.int/healthinfo/systems/surveydata/index.php/catalog/94
**Namibia**	DHS	2013	5548	1940	Have you ever had a test or exam to see if you have cervical cancer?	N/A	N/A	https://dhsprogram.com/pubs/pdf/FR298/FR298.pdf	https://dhsprogram.com/methodology/survey/survey-display-363.cfm
**South Africa**	WHS	2003	653	142	Asked to those who've ever had a pelvic exam within the past 3 years: The last time you had the pelvic examination, did you have a Pap smear test? (By Pap smear test, I mean did a doctor or nurse use a swab or stick to wipe from inside your vagina, take a sample and send it to a laboratory?)	N/A	N/A	N/A	https://apps.who.int/healthinfo/systems/surveydata/index.php/catalog/71
**South Africa**	SAGE	2007	176	67	Asked to those who ever had a pelvic exam: The last time you had the pelvic examination, did you have a Pap smear test? (By Pap smear test, I mean did a doctor or nurse use a swab or stick to wipe from inside your vagina, take a sample and send it to a laboratory?)	N/A	N/A	https://www.spirometry.com/wp-content/uploads/2020/02/South-Africa-WEB.pdf	https://apps.who.int/healthinfo/systems/surveydata/index.php/catalog/5
**South Africa**	SABSSM	2012	6424	3471	Have you ever had a test for a Pap smear? (By Pap smear test, I mean did a doctor or nurse use a swab or stick to wipe from inside your vagina, take a sample and send it to the laboratory).	When was the last time you had this test?	N/A	http://www.hsrc.ac.za/uploads/pageContent/4565/SABSSM%20IV%20LEO%20final.pdf	http://curation.hsrc.ac.za/index.php?module=pagesetter&type=user&func=hsrcdataset&ppnumber=PFAJLA&datasetno=30
**South Africa**	DHS	2016	2773	1225	Have you ever had a Pap smear? PROBE: When visiting a doctor or nurse, have you ever been asked to lie on your back with your legs apart so they could use a stick to take a sample from your vagina? The sample would have been sent to a laboratory for testing.	How many years ago was your last Pap smear?	N/A	https://dhsprogram.com/pubs/pdf/FR337/FR337.pdf	https://dhsprogram.com/methodology/survey/survey-display-390.cfm

**Zimbabwe**	WHS	2003	1337	125	Asked to those who've ever had a pelvic exam within the past 3 years: The last time you had the pelvic examination, did you have a Pap smear test? (By Pap smear test, I mean did a doctor or nurse use a swab or stick to wipe from inside your vagina, take a sample and send it to a laboratory?)	N/A	N/A	N/A	https://apps.who.int/healthinfo/systems/surveydata/index.php/catalog/69
**Zimbabwe**	DHS	2015	6017	1198	Have you ever been screened for cervical cancer?	When were you last screened for cervical cancer?	N/A	https://dhsprogram.com/pubs/pdf/FR322/FR322.pdf	https://dhsprogram.com/methodology/survey/survey-display-475.cfm
**Zimbabwe[a, b]**	PHIA	2020	1439	526	Have you ever been tested for cervical cancer?	What month and year was your last test for cervical cancer?	Did you receive treatment after your last test for cervical cancer? Did you receive treatment on the same day or on a different day?	https://phia.icap.columbia.edu/wp-content/uploads/2022/01/210122_ZIMPHIA2020-interactive-versionFinal.pdf	https://phia.icap.columbia.edu/zimbabwe2020-final-report/
**Zimbabwe**	PHIA	2015-16	6784	1129	Have you ever been tested for cervical cancer?	What month and year was your last test for cervical cancer?	N/A	https://phia.icap.columbia.edu/wp-content/uploads/2020/02/ZIMPHIA-Final-Report_integrated_Web-1.pdf	https://phia-data.icap.columbia.edu/datasets?country_id=6
**Western Africa**									
**Benin**	STEPS	2015	1739	13	Have you ever had a screening test for cervical cancer, using any of these methods described above (i.e., VIA, pap smear or HPV test)?	N/A	N/A	https://extranet.who.int/ncdsmicrodata/index.php/catalog/107/download/1044	https://extranet.who.int/ncdsmicrodata/index.php/catalog/107
**Benin**	DHS	2017-18	4659	39	Est-ce qu'un médecin ou du personnel de santé vous a déjà fait un test de détection du cancer du col de l'utérus ?	Quand a eu lieu votre dernier test pour le cancer de l'utérus?	Avez-vous suivi un traitement pour le col de l'utérus ou avez-vous fait des visites de suivi à cause des résultats du test ?[c]	https://dhsprogram.com/pubs/pdf/FR350/FR350.pdf	https://dhsprogram.com/methodology/survey/survey-display-491.cfm
**Burkina Faso**	WHS	2003	1305	100	Asked to those who've ever had a pelvic exam within the past 3 years: The last time you had the pelvic examination, did you have a Pap smear test? (By Pap smear test, I mean did a	N/A	N/A	N/A	https://apps.who.int/healthinfo/systems/surveydata/index.php/catalog/20

					doctor or nurse use a swab or stick to wipe from inside your vagina, take a sample and send it to a laboratory?)				
**Burkina Faso[a]**	STEPS	2013	528	42	Have you ever had a screening test for cervical cancer, using any of these methods described above (i.e., VIA, pap smear or HPV test)?	N/A	N/A	https://extranet.who.int/ncdsmicrodata/index.php/catalog/318/download/2484	https://www.who.int/publications/m/item/2013-steps-country-report-burkina-faso
**Cameroon**	DHS	2018	7700	389	Est-ce qu'un médecin ou un autre professionnel de santé vous a déjà fait un test de détection du cancer du col de l'utérus ?	N/A	N/A	https://dhsprogram.com/pubs/pdf/FR360/FR360.pdf	https://dhsprogram.com/methodology/survey/survey-display-511.cfm
**Cape Verde**	STEPS	2019	1431	708	Have you ever had a screening test for cervical cancer, using any of these methods described above (i.e., VIA, pap smear or HPV test)?	N/A	Did you receive any treatment to your cervix because of your test result?	N/A	Data obtained from members of the Catalan Institute of Oncology
**Chad**	WHS	2003	1115	71	Asked to those who've ever had a pelvic exam within the past 3 years: The last time you had the pelvic examination, did you have a Pap smear test? (By Pap smear test, I mean did a doctor or nurse use a swab or stick to wipe from inside your vagina, take a sample and send it to a laboratory?)	N/A	N/A	N/A	https://apps.who.int/healthinfo/systems/surveydata/index.php/catalog/77
**Côte d'Ivoire**	WHS	2003	682	59	Asked to those who've ever had a pelvic exam within the past 3 years: The last time you had the pelvic examination, did you have a Pap smear test? (By Pap smear test, I mean did a doctor or nurse use a swab or stick to wipe from inside your vagina, take a sample and send it to a laboratory?)	N/A	N/A	N/A	https://apps.who.int/healthinfo/systems/surveydata/index.php/catalog/93
**Côte d'Ivoire**	DHS	2011-12	6063	68	Avez-vous déjà fait un test du col de l'utérus ?	N/A	N/A	https://dhsprogram.com/pubs/pdf/FR272/FR272.pdf	https://dhsprogram.com/methodology/survey/survey-display-311.cfm
**Ghana**	WHS	2003	1146	41	Asked to those who've ever had a pelvic exam within the past 3 years: The last time you had the pelvic examination, did you have a Pap smear test? (By Pap smear test, I mean did a doctor or nurse use a swab or stick to wipe from inside your vagina, take a sample and send it to a laboratory?)	N/A	N/A	N/A	https://apps.who.int/healthinfo/systems/surveydata/index.php/catalog/96
**Ghana**	SAGE	2007	314	17	Asked to those who ever had a pelvic exam: The last time you had the pelvic examination, did you have a Pap smear test? (By Pap smear test, I mean did a doctor or nurse use a swab or stick to wipe from inside your vagina, take a sample and send it to a laboratory?)	When was the last time you had a pelvic examination, if ever? (By pelvic examination, I mean when a doctor or	N/A	https://apps.who.int/healthinfo/systems/surveydata/index.php/catalog/6/download/1940	https://apps.who.int/healthinfo/systems/surveydata/index.php/catalog/6

						nurse examined your vagina and uterus?)			
**Mali**	WHS	2003	724	43	Asked to those who've ever had a pelvic exam within the past 3 years: The last time you had the pelvic examination, did you have a Pap smear test? (By Pap smear test, I mean did a doctor or nurse use a swab or stick to wipe from inside your vagina, take a sample and send it to a laboratory?)	N/A	N/A	N/A	https://apps.who.int/healthinfo/systems/surveydata/index.php/catalog/79
**Mauritania**	WHS	2003	1064	43	Asked to those who've ever had a pelvic exam within the past 3 years: The last time you had the pelvic examination, did you have a Pap smear test? (By Pap smear test, I mean did a doctor or nurse use a swab or stick to wipe from inside your vagina, take a sample and send it to a laboratory?)	N/A	N/A	N/A	https://apps.who.int/healthinfo/systems/surveydata/index.php/catalog/98
**Sao Tome and Principe**	STEPS	2019	878	211	Have you ever had a screening test for cervical cancer, using any of these methods described above (i.e., VIA, pap smear or HPV test)?	N/A	N/A	N/A	Data obtained from members of the Catalan Institute of Oncology
**Senegal**	WHS	2003	620	93	Asked to those who've ever had a pelvic exam within the past 3 years: The last time you had the pelvic examination, did you have a Pap smear test? (By Pap smear test, I mean did a doctor or nurse use a swab or stick to wipe from inside your vagina, take a sample and send it to a laboratory?)	N/A	N/A	N/A	https://apps.who.int/healthinfo/systems/surveydata/index.php/catalog/87
**Senegal**	STEPS	2014	1976	189	Have you ever had a screening test for cervical cancer, using any of these methods described above (i.e., VIA, pap smear or HPV test)?	N/A	N/A	https://www.ansd.sn/ressources/publications/DV-STEPS-1-06-2016%20-%20MF-fin_ANSD%20vf.pdf	https://demostaf.web.ined.fr/index.php/catalog/114/study-description

[a]Microdata unavailable thus sample size values were calculated using survey tabulations
[b]Only information for WLHIV is available
[c]Only 3 respondents answered this question thus data from this question was not used
[d]Represents number of women who self-report screening in their lifetime for surveys with this information, for surveys without lifetime screening information (i.e., WHS) represents number of women reporting screening in the past 3 years
[e]Informed consent for surveys were recorded through consent forms following verbal communication from the surveyors describing the purpose and scope of surveys. To protect the privacy and safety of participants, data is then anonymized, and researchers must request and be approved for use of data before access is granted to surveys. More specific details regarding consent procedures may be found in specific country reports

Text A. Description of the methods and additional results for regional and national estimates of cervical cancer screening coverage time trends

Screening Time Trends Model Equations

This section describes the model equations used to estimate the time trends in screening coverage for cervical cancer (CC). When HIV serostatus data was available, the model takes the following form:

$$Y_i \sim Binomial(N_i, p_i)$$

$$logit(p_i) = \alpha + \mu_{s[i]} + \upsilon_{c[i]} + \omega_{r[i]} + \delta_{r[i]} \times T_i + \sum_a (\beta_{a[i]} \times A_i) + \sum_a (\kappa_{a[i]} \times L_i) + \gamma_{c[i]} \times H_i$$

Where Y_i is the survey-adjusted number of women reporting CC screening for observation i (Table B), N_i is the effective sample size for that observation and p_i is the probability that women reported screening which was modelled on the logistic scale. CC screening coverage is modeled hierarchically with α as the global intercept, $\mu_{s[i]}$ as the survey-level intercepts, $\upsilon_{c[i]}$ as the country-level intercepts, and $\omega_{r[i]}$ as the region-level intercepts. We considered potential regional time trends through random slope coefficients $\delta_{r[i]}$ (i.e., linear trends on the logit scale) and T_i as the centered calendar time in years. Age is modeled as a vector of fixed effects $\beta_{a[i]}$ using five categories (25-29, 30-34, 35-39, 40-44, and 45-49 years) that correspond to the age group of the observation using indicator variable A_i. As the model included CC screening outcomes for both lifetime and past three years, we use age-specific indicators $\kappa_{a[i]}$ (as fixed effects) to "*cross-walk*" between the two recall periods[14], with L_i being a dummy variable indicating if the outcome for observation *i* corresponds to the three-year recall period. Finally, any potential effect of living with HIV on CC screening coverage is considered using country-level ($\gamma_{c[i]}$) random slope coefficients with H_i being an indicator of HIV status. This country-level random effect for HIV is nested within an overall and region-level effect ($\gamma_{c[i]} = \rho_{h[i]} + \rho_{r[i]} + \rho_{c[i]}$)

When HIV serostatus is not available, standardization is performed, and takes the following form:

$$p_i = \widehat{HIV}_{c[i]} \times logit^{-1}\left(\alpha + \mu_{s[i]} + \upsilon_{c[i]} + \rho_{r[i]} + \delta_{r[i]} \times T_i + \sum_a (\beta_{a[i]} \times A_i) + \sum_a (\kappa_{a[i]} \times L_i) + \gamma_{r[i]}\right) +$$

$$(1\text{-}\widehat{HIV}_{c[i]}) \times logit^{-1}\left(\alpha + \mu_{s[i]} + \upsilon_{c[i]} + \rho_{r[i]} + \delta_{r[i]} \times T_i + \sum_a (\beta_{a[i]} \times A_i) + \sum_a (\kappa_{a[i]} \times L_i)\right)$$

Where $\widehat{HIV}_{c[i]}$ represents the country and year-specific HIV prevalence among women 25-49 years, derived from UNAIDS data.

The model specification is completed using weakly informative prior distributions (see below). Posterior distributions of the parameters of interest are obtained using Hamiltonian Monte Carlo simulations. Specifically, inferences are based on six chains of 3,000 iterations. Convergence was examined using trace plots and by ensuring that the potential scale reduction factor for all parameters and hyperparameters remained close to one[1].

Model Priors

Priors for the global, regional, country, and survey-level intercepts

The Bayesian model specification presented in the manuscript is completed using the following prior distributions.

$$\alpha \sim \mathcal{N}(0,\ 5)$$
$$\omega_r \sim \mathcal{N}(0,\ \sigma_r) \quad and \quad \sigma_r \sim \mathcal{HC}(0, 3)$$
$$\upsilon_c \sim \mathcal{N}(0,\ \sigma_c) \quad and \quad \sigma_c \sim \mathcal{HC}(0, 3)$$
$$\mu_s \sim \mathcal{N}(0,\ \sigma_s) \quad and \quad \sigma_s \sim \mathcal{HC}(0, 3)$$

Where α represents the global intercept, ω_r represents region-level random intercepts, υ_c represents country-level, and μ_s is the survey-level random intercept. Each random intercept follows a normal distribution with mean 0 and has its own standard deviation (i.e., σ_r, σ_s, and σ_s). These standard deviations are given weakly informative half-Cauchy ($\mathcal{HC}$) priors, in line with recommendations[1].

Priors for the region-level random slope for time

$$\eta_t \sim N(0,10)$$
$$\delta_r \sim \mathcal{N}(\eta_t,\ \sigma_t) \quad and \quad \sigma_t \sim \mathcal{HC}(0,3)$$

Where η_t is the overall time trend (i.e., the log-odds ratio for time) and δ_r is the region-level time trend. The degree of pooling between the different regions' trends is determined by the standard deviation σ_t which is given a half-Cauchy prior.

Priors of the fixed effects for age and recall period

$$\beta_a \sim \mathcal{N}(0,\ 5)$$
$$\kappa_a \sim \mathcal{N}(0,\ 5)$$

Where β_a represents the fixed effect for each 5-year age group *a* and κ_a is the vector of fixed effects for the recall period (i.e., lifetime versus past three years) for each 5-year age group *a*.

Priors of the overall, region- and country-level random slopes for HIV

$$\rho_h \sim N(0,10)$$
$$\rho_r \sim \mathcal{N}(0,\ \pi_r) \quad and \quad \pi_r \sim \mathcal{HC}(0,3)$$
$$\rho_c \sim \mathcal{N}(0,\ \pi_c) \quad and \quad \pi_c \sim \mathcal{HC}(0,3)$$

Where ρ_h is the overall pooled log-odds ratio for the effect of HIV on cervical cancer (CC) screening, ρ_r contains the region-level random slopes, and ρ_c the country-level random slopes for HIV. The standard deviations for the region-level (π_r) and the country-level (π_c) random slopes are given half-Cauchy priors.

Imputations and post-stratification

The Bayesian multilevel model provides estimates of the many parameters for the global, regional, and country-level intercepts. When combined with the regional random slopes for time, the fixed effects for age, the recall period, and HIV status, we can estimate screening coverage ($\hat{p}_{c,a,t,r,h}$) for all relevant strata for country (*c*), age (*a*), time (*t*), recall period (*r*), and HIV status (*h*) using the equation below

$$logit(\hat{p}_{c,a,t,r,h}) = \hat{\alpha} + \hat{\rho}_{r[c]} + \hat{\mu}_c + \hat{\delta}_{r[c]} \times T_t + \sum_a (\hat{\beta}_a \times A_a) + \sum_a (\hat{\kappa}_a \times L_a) + \hat{\gamma}_c \times H_h$$

In instances where we needed to predict a country without any survey data, we imputed their coverage based on the regional coverage and added the country-level uncertainty by sampling from the distribution of country-level random effects (i.e., $\upsilon_c \sim \mathcal{N}(0,\ \sigma_c)$). Similarly, if a country did not have HIV information, coverage among WLHIV was estimated from the region-level random slope and the country-level uncertainty sampled from the distribution of the country-level random slopes.

The overall aggregate value for proportion of women screened was obtained by summing the proportion of women screened per age group and HIV status for each draw from the posterior multiplied by a weighted value for the specific age group, HIV status, and country using *the UN World Population Prospects* data and UNAID-derived HIV prevalence estimates. This value is then divided by the overall sum to obtain our aggregate estimate.

The results for countries with two or more surveys are presented in Fig A and Fig B. Regional and country-level estimates for 2020 are presented in Table C.

Model validation for regional and national estimates of cervical cancer screening coverage
Several candidate models were evaluated prior to selection of the final model. These candidate models include a) different nested structures for the random effects, b) addition of a time-varying fixed effect indicating whether a national screening program was implemented, and c) using *Gross National Income* (GNI) per capita as a time-varying fixed effect. Ultimately, the model without an effect for national screening programs was preferred due to the limited amount of high-quality data regarding the actual implementation of those programs and limited impact on model fit. Similarly, adding GNI per capita did not influence our results (Fig C and Fig D). Additionally, a sensitivity analysis was done to investigate the impact of utilizing only surveys conducted after 2010 (Fig E). Overall, the general qualitative trends and low screening coverages remain the same. However, there is a slight change in the direction of the trend for Western/Central Africa, reflecting the lack of repeated surveys in that region. In a second sensitivity analysis, we included a fixed-effect in the model to adjust for the *World Health Survey* (WHS) –the most common survey conducted in the early 2000s. Results of this analysis saw minimal changes from those of our main analysis (Fig E).

The posterior predictive checks (Fig F), where model predictions are compared to the empirical observations, suggested that the current model fit the data well and that differences, if any, were small and within uncertainty intervals. Finally, in-sample comparisons suggest small prediction errors with median absolute errors around 2% and reasonable coverage of credible intervals (Table D).

A

B

C

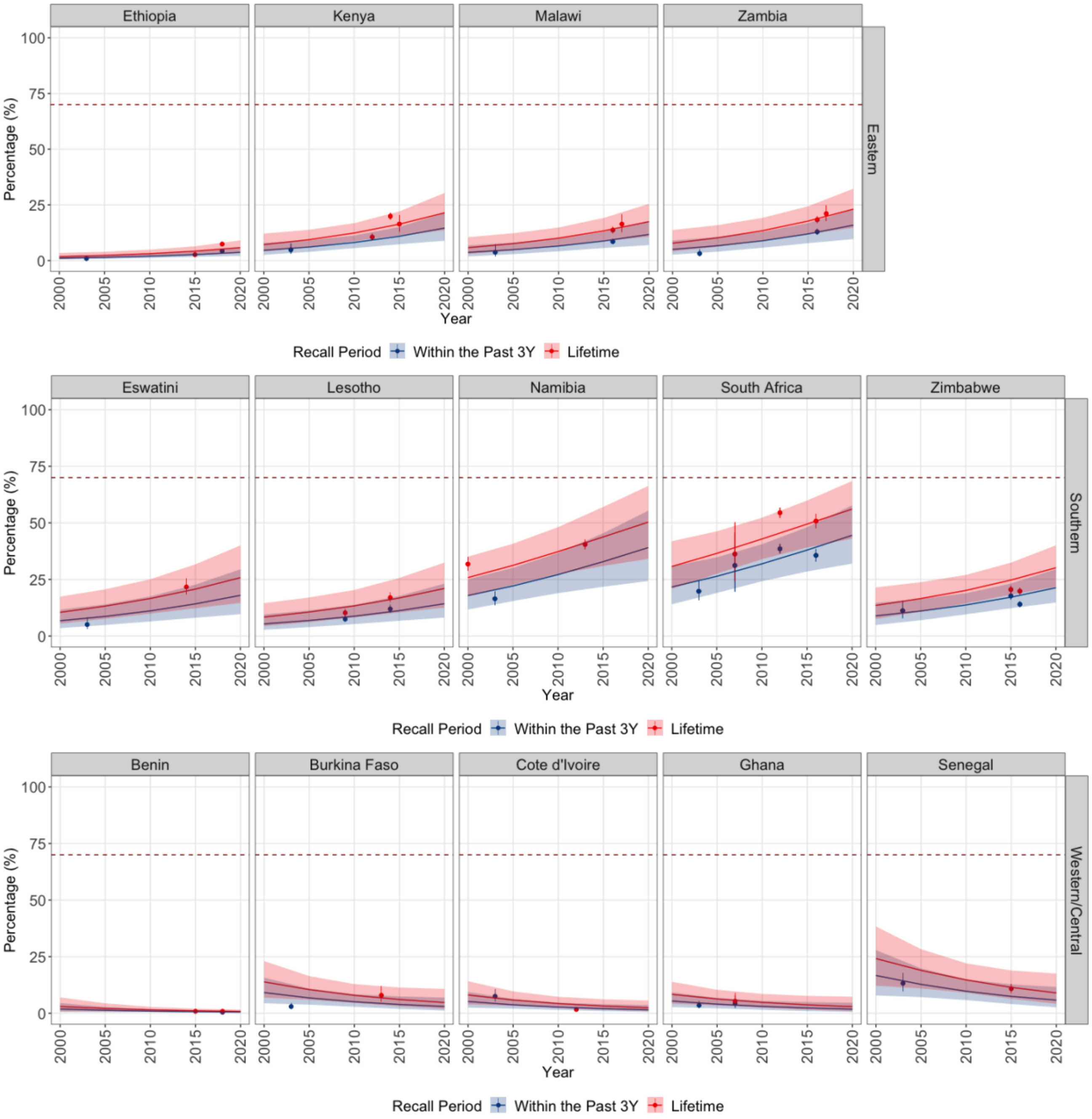

Fig A. Country-level trends in lifetime and past three-year cervical cancer screening coverage among women aged 30-49 years between 2000-2020.
Panel A) Eastern African countries. Panel B) Southern African countries. Panel C) Western/Central African countries. The red trendline represents screening trends for lifetime screening. The blue trendline represents screening trends for screening in the past three years. Points represent the empirical survey estimates of women reports of having been screened for cervical cancer. The dotted red line represents the 70% screening goal set by the *World Health Organization*.

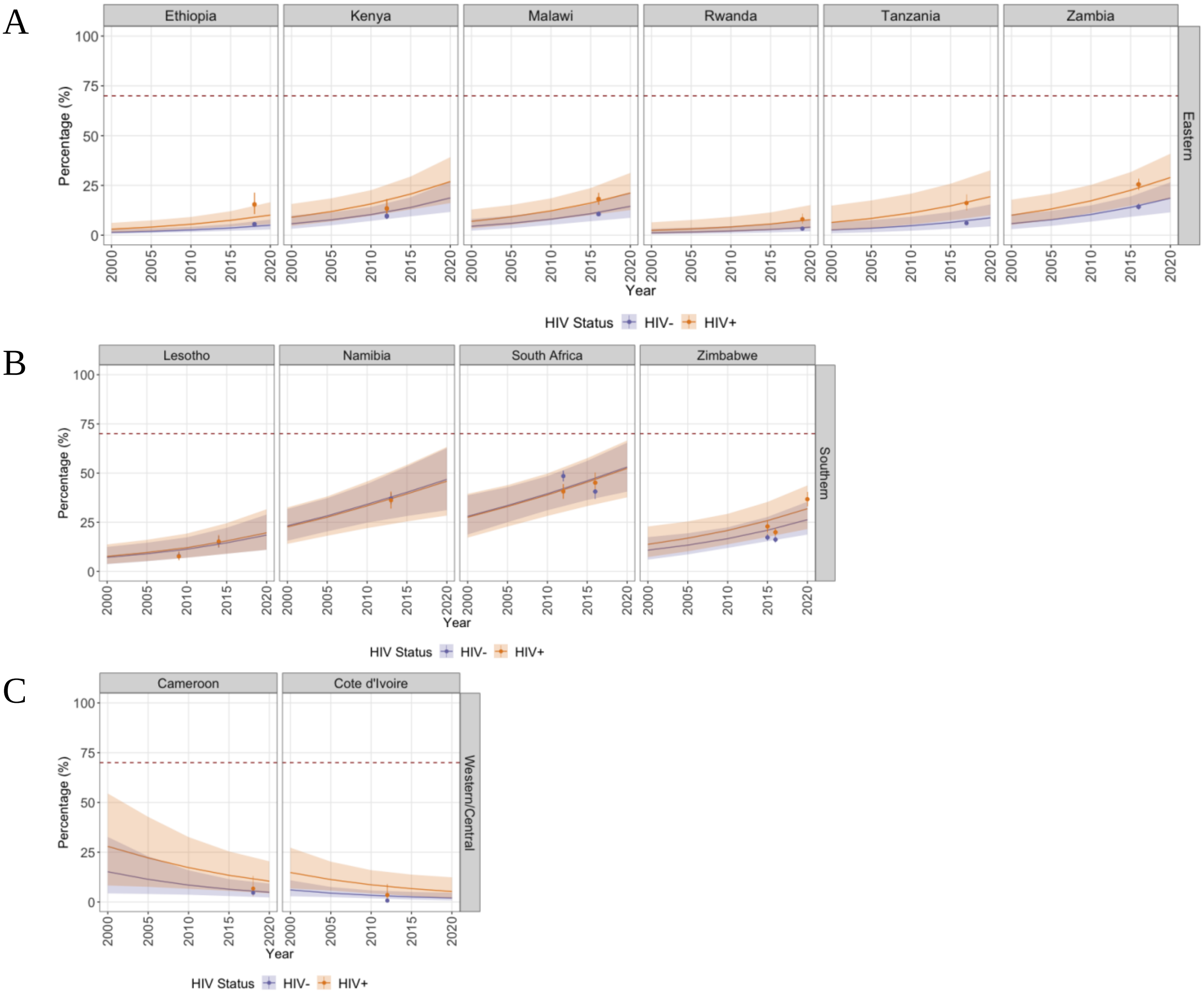

Fig B. Country-level trends of lifetime cervical cancer screening stratified by HIV status for women aged 25-49 years between 2000-2020.

Panel A) Eastern African countries. Panel B) Southern African countries. Panel C) Western/Central African countries. The purple trendline represents screening trends for women living with HIV. The orange trendline represents screening trends for women without HIV. Points represent weighted screening proportions directly from survey data stratified by HIV status. The dotted red line represents the 70% screening goal set by the *World Health Organization*.

Table C. Regional and country-level estimates of the percentage of women screened for cervical cancer in their lifetime and in the past three years in 2020 for countries with two or more surveys by five-year age groups between 30-49 years.

	Lifetime screening (95%CrI)			
	**30-34 years**	**35-39 years**	**40-44 years**	**45-49 years**
**Overall**	12% (9-19%)	14% (11-21%)	15% (12-23%)	15% (12-24%)
**Western/Central Africa**	5% (2-18%)	6% (2-21%)	7% (3-23%)	7% (3-23%)
Benin	1% (0-1%)	1% (0-2%)	1% (0-2%)	1% (0-2%)
Burkina Faso	3% (2-9%)	4% (2-11%)	5% (2-12%)	5% (2-12%)
Côte d'Ivoire	2% (1-5%)	2% (1-6%)	2% (1-6%)	2% (1-6%)
Ghana	2% (1-6%)	2% (1-7%)	3% (1-8%)	3% (1-8%)
Senegal	7% (3-15%)	8% (4-17%)	9% (5-20%)	9% (5-20%)
**Eastern Africa**	11% (8-17%)	13% (9-19%)	15% (11-21%)	15% (11-21%)
Ethiopia	5% (3-8%)	6% (3-9%)	6% (4-10%)	6% (4-10%)
Kenya	18% (11-26%)	21% (14-31%)	24% (15-34%)	24% (15-34%)
Malawi	15% (9-22%)	17% (11-26%)	20% (12-28%)	20% (12-28%)
Zambia	20% (12-28%)	23% (15-32%)	26% (17-36%)	26% (17-36%)
**Southern Africa**	46% (35-57%)	51% (40-62%)	54% (43-65%)	55% (44-66%)
Eswatini	22% (12-35%)	25% (15-40%)	28% (17-44%)	28% (16-43%)
Lesotho	17% (11-28%)	21% (13-33%)	23% (14-36%)	23% (14-36%)
Namibia	45% (30-62%)	51% (34-67%)	54% (37-70%)	54% (38-70%)
South Africa	51% (38-64%)	56% (43-69%)	60% (47-72%)	60% (47-72%)
Zimbabwe	26% (19-36%)	30% (22-40%)	33% (24-44%)	33% (24-44%)
	**Screening in the past three years (95%CrI)**			
	**30-34 years**	**35-39 years**	**40-44 years**	**45-49 years**
**Overall**	9% (7-14%)	10% (8-16%)	11% (8-17%)	10% (7-16%)
**Western/Central Africa**	4% (1-14%)	4% (2-15%)	5% (2-16%)	4% (2-15%)
Benin	0% (0-1%)	1% (0-1%)	1% (0-1%)	0% (0-1%)
Burkina Faso	2% (1-6%)	3% (1-7%)	3% (1-8%)	3% (1-7%)
Côte d'Ivoire	1% (1-3%)	1% (1-4%)	1% (1-4%)	1% (1-4%)
Ghana	1% (1-4%)	2% (1-5%)	2% (1-5%)	2% (1-5%)
Senegal	5% (2-11%)	5% (3-12%)	6% (3-13%)	5% (3-11%)
**Eastern Africa**	8% (6-13%)	9% (6-14%)	10% (7-15%)	9% (6-14%)
Ethiopia	3% (2-5%)	4% (2-6%)	4% (2-6%)	4% (2-6%)
Kenya	13% (8-20%)	15% (9-22%)	16% (10-23%)	14% (9-21%)
Malawi	11% (6-17%)	12% (7-18%)	13% (8-19%)	11% (7-18%)
Zambia	15% (9-22%)	16% (10-24%)	17% (11-25%)	15% (10-23%)
**Southern Africa**	37% (27-49%)	40% (30-52%)	42% (31-53%)	40% (29-51%)
Eswatini	16% (9-28%)	18% (10-30%)	19% (10-32%)	17% (9-29%)
Lesotho	13% (8-22%)	14% (8-24%)	15% (9-25%)	14% (8-23%)
Namibia	37% (23-53%)	40% (25-56%)	41% (26-58%)	38% (24-55%)
South Africa	42% (30-56%)	45% (33-59%)	47% (34-60%)	44% (31-57%)
Zimbabwe	20% (14-28%)	22% (15-30%)	23% (16-32%)	21% (14-29%)

95%CrI = 95% credible intervals.

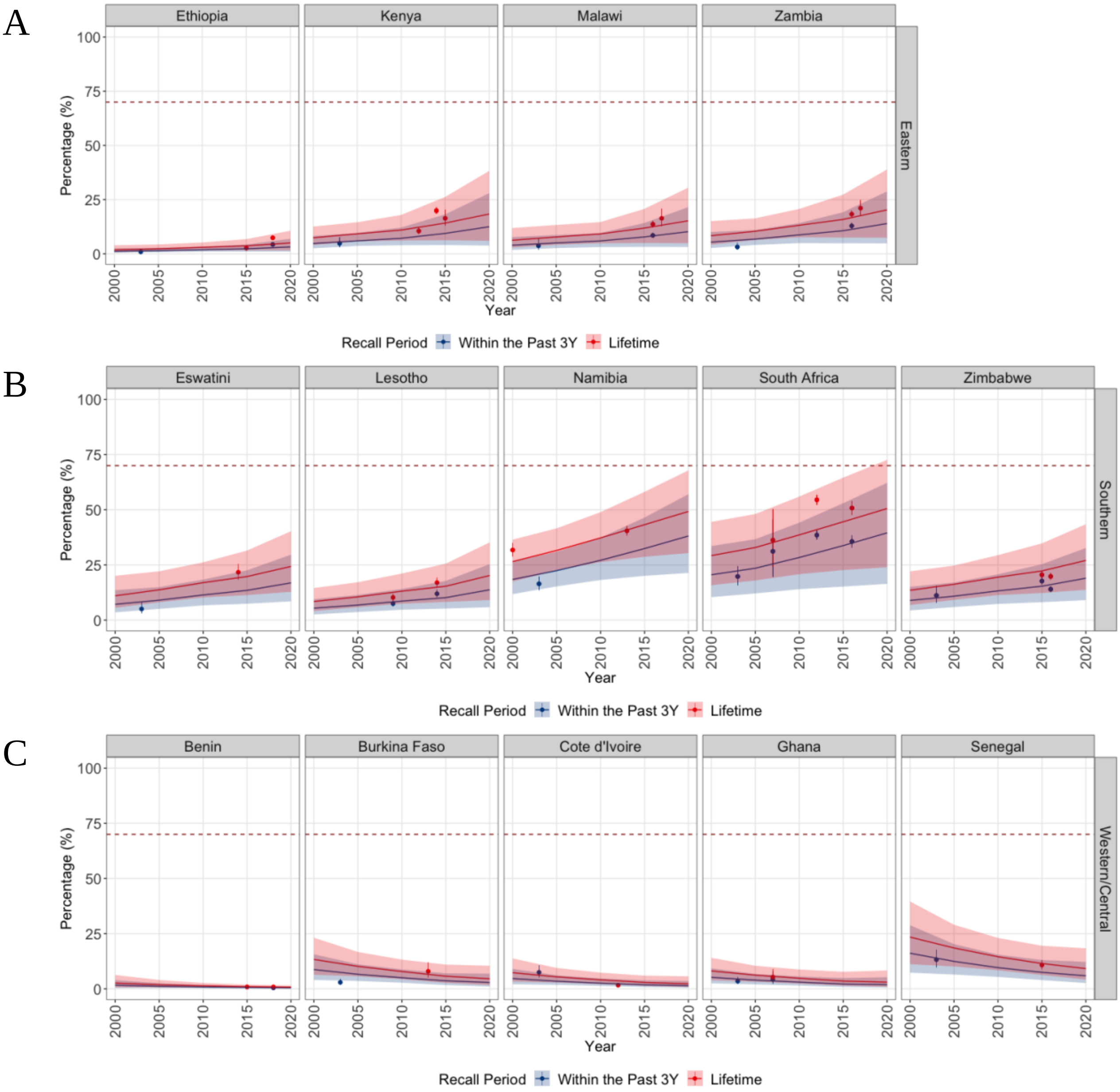

Fig C. Sensitivity analysis of country-level screening time trends with a fixed effect for national screening program. Panel A) Eastern African countries. Panel B) Southern African countries. Panel C) Western/Central African countries. Circles represent the survey estimates. Lines represent the median estimates and the shaded areas their 95% credible intervals.

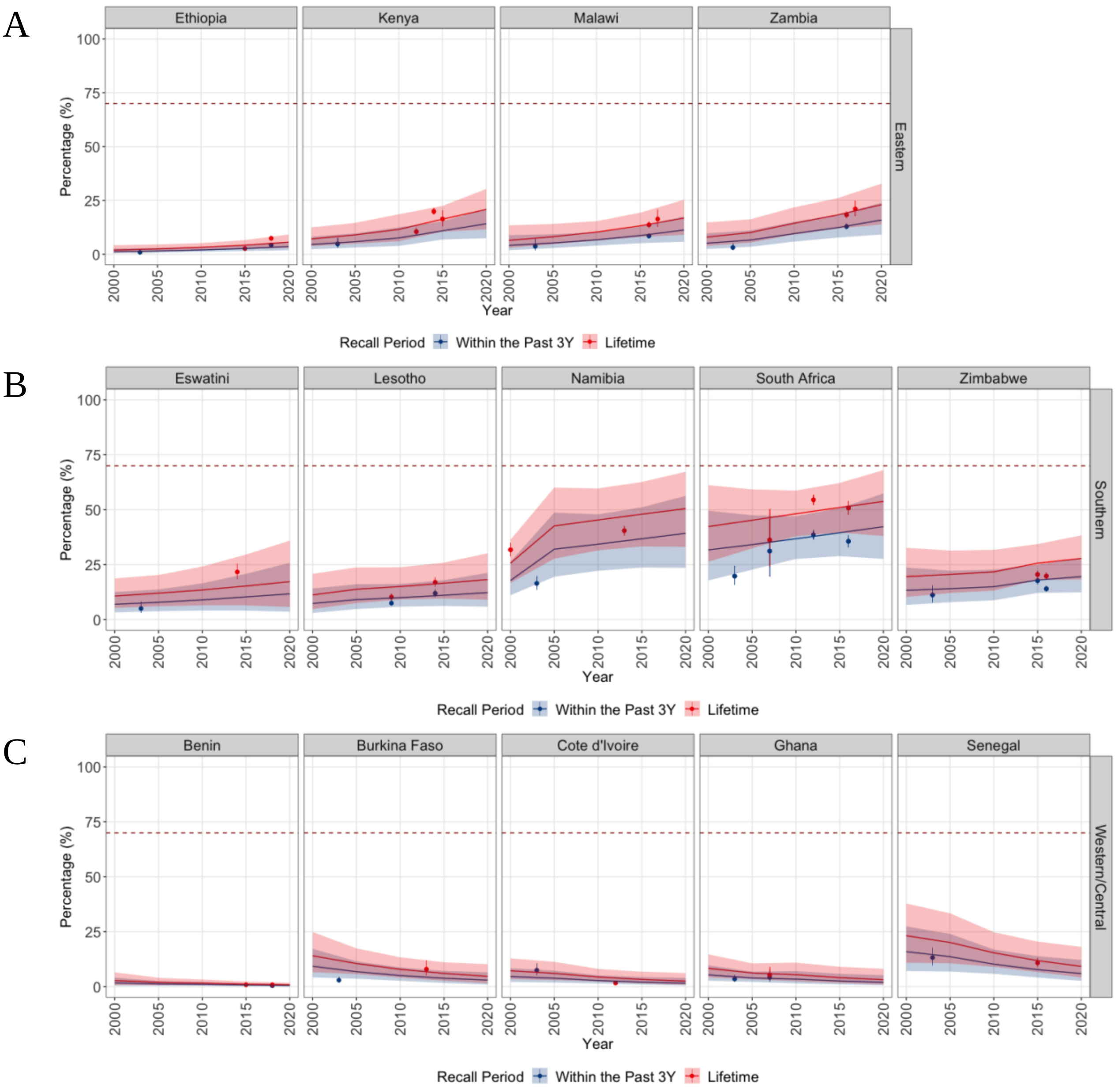

Fig D. Sensitivity analysis of country-level screening time trends with a fixed effect for Gross National Income. Panel A) Eastern African countries. Panel B) Southern African countries. Panel C) Western/Central African countries. Circles represent the survey estimates. Lines represent the median estimates and the shaded areas their 95% credible intervals. The dotted red line represents the 70% screening goal set by the *World Health Organization*.

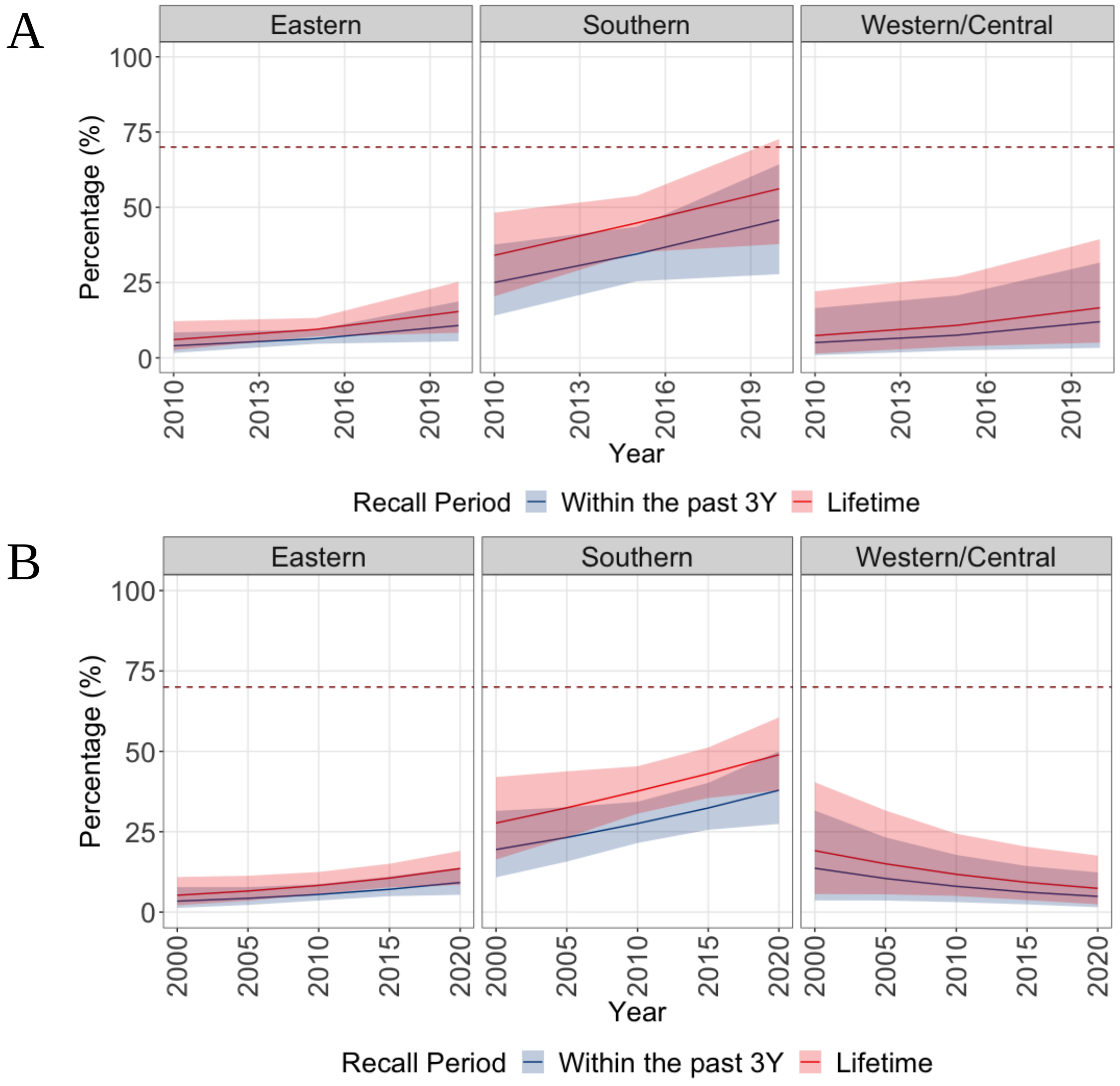

Fig E. Sensitivity analyses of regional-level trends in lifetime and past three years cervical cancer screening coverage among women aged 30-49 years.
Panel A) Using survey data obtained only between years 2010-2020. Panel B) Using all data but including a dummy variable in the model to adjust for a potential effect of the World Health Surveys (WHS).
The red trendline represents lifetime screening trends. The blue trendline represents screening trends for screening in the past three years. Shaded regions represent the 95% credible intervals. The dotted red line represents the 70% screening goal set by the *World Health Organization*.

A

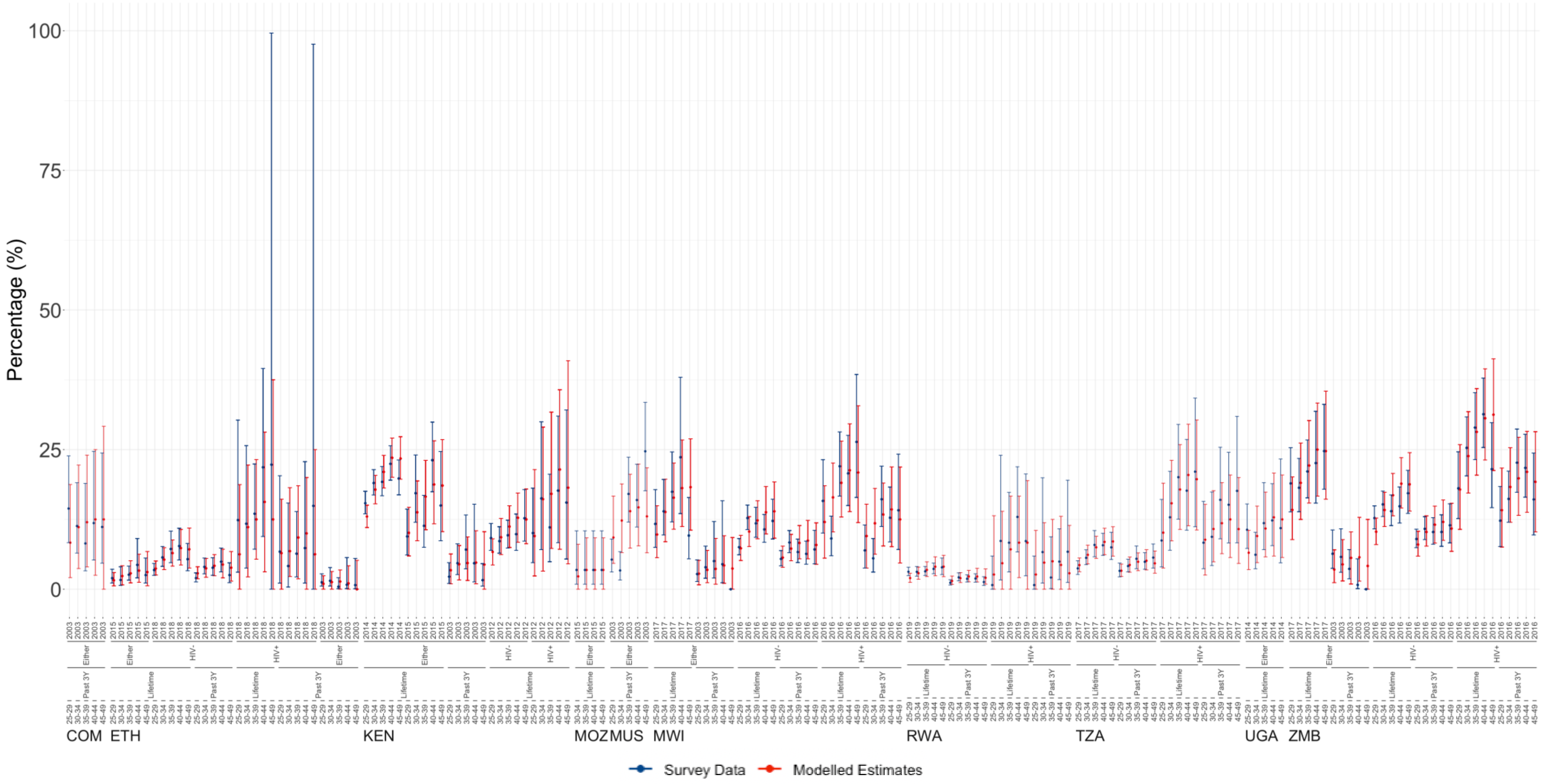
Percentage (%)
100
75
50
25
0
COM
ETH
KEN
MOZ
MUS
MWI
RWA
TZA
UGA
ZMB
Survey Data
Modelled Estimates

B

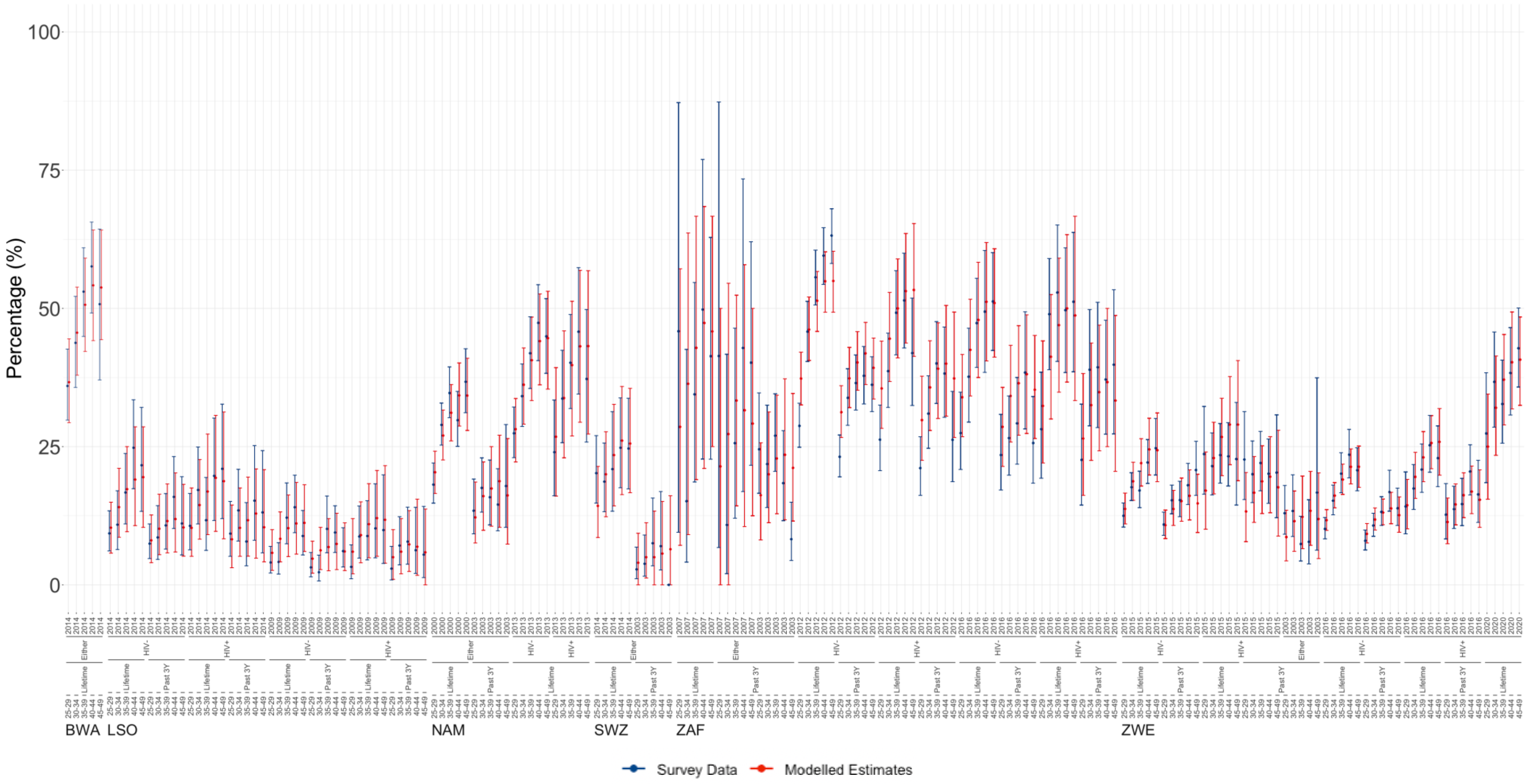
Percentage (%)
100
75
50
25
0
BWA
LSO
NAM
SWZ
ZAF
ZWE
Survey Data
Modelled Estimates

C

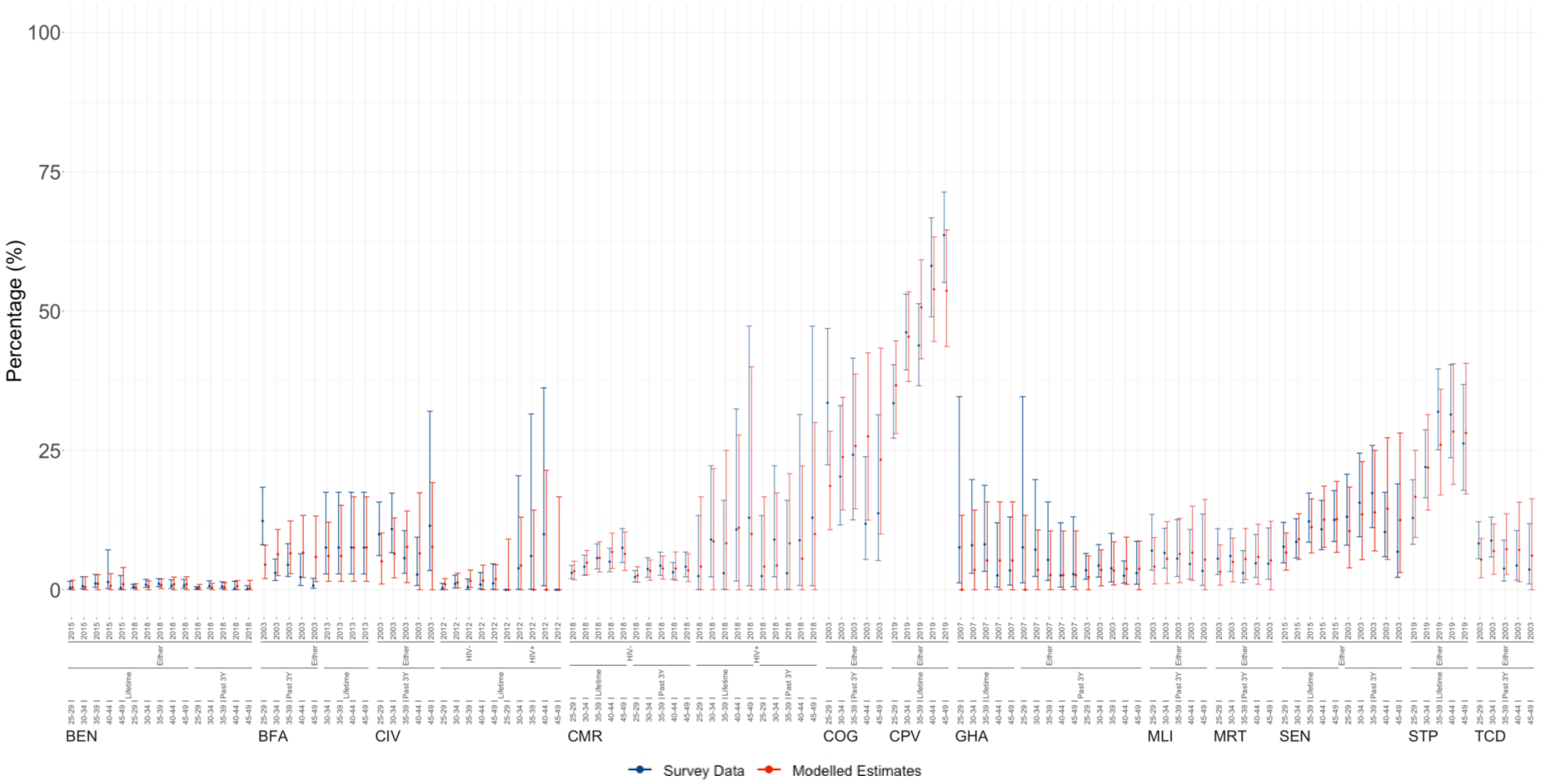

Fig F. Posterior predictive checks comparing modelled estimates (red) to each empirical data point (blue) for the three regions.
Panel A) Countries in the Eastern Africa region. Panel B) Countries in the Southern Africa region. Panel C) Countries in the Western/Central Africa region. The circles represent the values of the observations or of the median estimates. The errors bars represent the 95% confidence interval of the survey data or the 95% credible intervals of the modeled estimates. The three

Table D. In-sample comparisons of model fits with empirical survey observations.

Recall period	Median error (%)	Median absolute error (%)	Below 95% CrI (%)	Above 95% CrI (%)
Within the past three years	0.1	2.4	6.4	3.2
Lifetime	-0.5	2.1	3.6	2.6
Overall	-0.3	2.2	4.9	2.9

CrI = credible interval.
The median error represents the median value for observed data minus the modeled estimates.
The median absolute error represents the median value of the absolute difference between observations and modeled estimates.
The assess coverage of our uncertainty estimates, we calculated the proportion of data points above and below the 95%CrI of the estimates.

Text B. Description of the methods and additional results for the WHO recommendations for frequency of re-screening.

Life table method to estimate the number of times women have been screened

The conceptual overview of the methods used to estimate the re-screening ratios and the proportion screened twice by age 45 is presented in Fig G. Age- and country-specific estimates of screening rates were obtained for every calendar year over 2005-2020 from the Bayesian multilevel model from the first objective (Text A). Specifically, we used estimates of the proportion screened in the past three years for each country, year, and five-year age group and converted that to an annual screening rate. This conversion assumed that screening rates were exponentially distributed (i.e., *rate = -(log(1-risk))/time*) and that it was an overall rate (i.e., includes first-time screen and repeated screens). This overall rate was then converted into re-screening and first-time screening rates using a modelled rate ratio between the rate of re-screening and first-time screening. Using life table methods, we then subjected a cohort of women aged 30 years to the age-, country-, and year-specific rates of first-time screening and re-screening (similar to calculations of life expectancy). The model was initialized in 2005 with women entering the life table as either never screened or screened once by the age of 30. The number of women who entered each category was informed by the Bayesian multilevel model from the first objective, specifically the proportion of women who had ever been screened between the ages of 25-29 years. At each time step (Δt = 0.1 year), never screened women could receive their first CC screen at an age and country-specific first-time screening rate. Those that had already been screened can be re-screened again, albeit at a different rate. This re-screening rate was informed by our analyses of the re-screening rate ratio (next section). At the end of each calendar year, the cohort of women was aged by one year, and age-specific rates of first-time screening and re-screening updated. We do not consider mortality as we assumed that, besides CC mortality, background mortality rates should not differ as a function of screening status. We proceeded as such for the next 15 years when this cohort of women would have reached the age of 45 years. Uncertainty was considered by using draws from the posterior distributions of the estimates (i.e., screening rates and re-screening ratios) to obtain the proportion of women screened twice by the aged of 45. The life table can be modelled using the following difference equations:

$$S^0_{a,t} = S^0_{a,t-1} - \Delta t(\lambda_{a,t} \times S^0_{a,t-1})$$

$$S^1_{a,t} = S^1_{a,t-1} + \Delta t(\lambda_{a,t} \times S^0_{a,t-1} - \phi \times \lambda_{a,t} \times S^1_{a,t-1})$$

$$S^2_{a,t} = S^2_{a,t-1} + \Delta t(\phi \times \lambda_{a,t} \times S^1_{a,t-1})$$

Where $S^0_{a,t}$ represents the number of women age a never screened at time t, $S^1_{a,t}$ represents the number of women age a screened once at time t, and $S^2_{a,t}$ represents the number of women age a screened twice or more at time t. Here, λ_a represents the first-time screening rate for women at age a (one of three 5-year age groups between 30-45 years). Finally, ϕ is the rate ratio between the rate of re-screening and first-time screening.

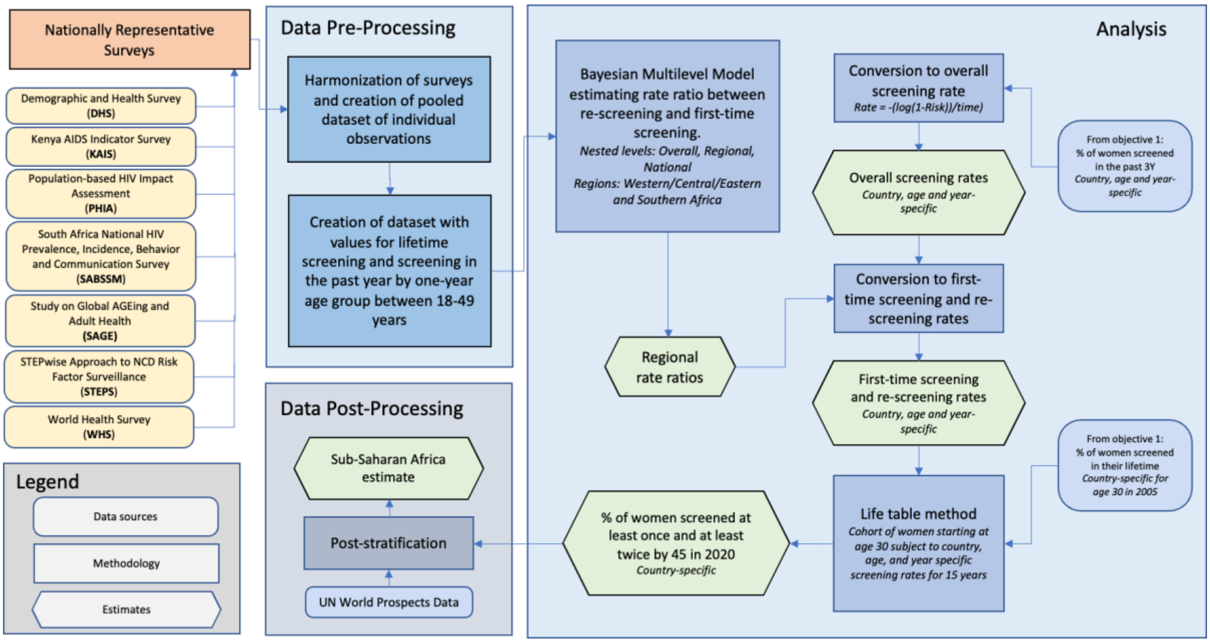

Fig G. Conceptual framework to estimate re-screening rates and screening twice in a lifetime by age 45 outlining data inputs, data pre-processing, statistical analyses, and data post processing. UN=United Nations.

Estimation of the re-screening rate ratio

To obtain rates of first-time screening and re-screening, a Bayesian model was employed to estimate a re-screening rate ratio. This rate ratio was estimated from cross-sectional survey data where we abstracted information on lifetime and past year screening coverage for one-year age groups. The data is thus composed of the proportion of women reporting having ever been screened and the proportion screened in the last year for each survey and for each one-year age group. From this data, we estimated two key parameters: i) the age-specific rate of first screening and ii) the rate ratio for re-screening. The equations for this model are the following:

$$p_{s,a}^{ever} = P_{s,a-1}^{ever} + \lambda_{s,a} \times P_{s,a-1}^{never}$$
$$p_{s,a}^{past} = \left(\left(\lambda_{s,a} \times P_{s,a-1}^{never} \right) + \phi_s \times \lambda_{s,a} \times P_{s,a-1}^{ever} \right) \times \tau$$

These equations state that the proportion of women ever screened in survey s for age a ($p_{s,a}^{ever}$) is the sum of the survey estimates of the proportion of women ever screened in the previous age group ($P_{s,a-1}^{ever}$) and the product of the survey- and age-specific first-time screening rate ($\lambda_{s,a}$) and the survey estimates of women who have never been screened in the previous age group ($P_{s,a-1}^{never}$). Similarly, the proportion of women screened in the past year for survey s and age a ($p_{s,a}^{past}$) is the sum of the proportion of women who are screened for the first time ($\lambda_{s,a} \times P_{s,a-1}^{never}$) and the proportion who have been re-screened ($\phi \times \lambda_{s,a} \times P_{s,a-1}^{ever}$). Here, ϕ_s represents the survey-specific rate ratio between first-time screening and re-screening. The ϕ_s parameter was assumed to be age and time-invariant. Further, it was not possible to adjust for the age and HIV status since the denominators for WLHIV by one-year age group would have been too small to be reliable. Finally, we adjusted for potential telescoping bias using the τ parameter in sensitivity analyses. Simulations were done to validate this model and understand under which conditions it could produce unbiased estimates of the rate ratio (see next section). The model was fit to the survey data using the following binomial likelihoods:

$$Y_{s,a}^{ever} \sim Binomial\left(N_{s,a}^{ever},\ p_{s,a}^{ever}\right)$$
$$Y_{s,a}^{past} \sim Binomial\left(N_{s,a}^{past},\ p_{s,a}^{past}\right)$$

Where $Y_{s,a}^{ever}$ is the survey-adjusted number of women reporting have ever been screened for CC in survey s and age group a, $N_{s,a}^{ever}$ is the survey denominator, $Y_{s,a}^{past}$ is the survey-adjusted number of women reporting have been screened in the past year, and $N_{s,a}^{past}$ is the denominator.

Priors used for this model were weakly informative.

$$\log(\lambda_{s,a}) \sim N(\log(0.005),\ 5)$$
$$\log(\phi_s) \sim N\big(\log(\phi_{r[s]}),\ \vartheta_{r[s]}\big)$$
$$\log(\phi_{r[s]}) \sim N(\log(\phi_o),\ \vartheta_o)$$
$$\log(\phi_o) \sim N(0,\ 5) \quad \text{and} \quad \vartheta_o \sim HC(0,\ 5)$$
$$\vartheta_{r[s]} \sim HC(0,\ 5)$$

Where $\lambda_{s,a}$ represents the survey and age specific rates of first-time screening. ϕ_s represents the survey-specific rate ratio between re-screening and first-time screening, ϕ_r represents the region-specific rate ratio (i.e., Western/Central/Eastern Africa or Southern Africa), and ϕ_o represents the overall rate ratio for sub-Saharan Africa. Western, Central, and Eastern Africa were modelled together as there was limited data on screening in the past year from surveys in Western/Central Africa. The survey-specific rate ratios are assumed to be distributed across a normal distribution with the mean as the overall region-specific rate ratio, and a region-specific standard deviation (ϑ_r). Similarly, the region-specific rate ratios are distributed across a normal distribution with the mean as the overall rate ratio for sub-Saharan Africa, and standard deviation ϑ_o

Our results found that the survey-specific rate ratios estimated by the model were highly variable, but consistently above one (Table E). As a result, the region-specific rate ratios were used to calculate the age-specific first-time screening and re-screening rates to be used in the life table.

Simulations to validate the proposed approach

To validate the approach for the estimation of re-screening rate ratios using cross-sectional survey data, we investigated the impact of age, cohort effects and period effects using simulations (Fig H). Simulations were done to mimic the levels of lifetime screening found from the first objective and was done using two simulated regions with three countries each. Estimates for the rate ratio for the two regions were jointly modelled. First simulations were performed using screening rates that varied with age but were assumed stable throughout time (i.e., no cohort effects). The model predicted the "true" simulated estimates well under these conditions (Fig I). Simulations were then performed using screening rates that differed with age and changed with time (i.e., cohort effects). These simulations found that the model only reproduced the "true" simulated results when the cohort effects were weak. When screening rates increased significantly throughout time however, the model would overestimate the rate ratios (ϕ_s) and these cohort effects would bias our results (Fig I). However, these biases could be minimized when restricting the analyses to younger age groups (Fig I). Finally, simulations were performed using screening rates that remained constant through time apart from a three-year period where screening was elevated proportionately for all age groups (e.g., period effects). In the presence of a weak period effect, our model was able to predict the true estimates relatively well. However, stronger period effects led to greater underestimation of the rate ratios (Fig I).

Previous analyses of the time trends of screening coverage suggest that cohort effects could be absent or small in countries within Western/Central and Eastern Africa as screening coverage was found to be relatively stagnant over time. However, this was not the case for Southern Africa, as such we only used data from women 18-29 years in this region. In contrast, we used all data from women aged 18-49 years in Western/Central/Eastern Africa. Age groups start at age 18 as that is the age in which all available surveys have data. Additionally, this analysis assumes that period effects are minimal and that screening services were available in sub-Saharan Africa for at least 30 years prior to 2020.

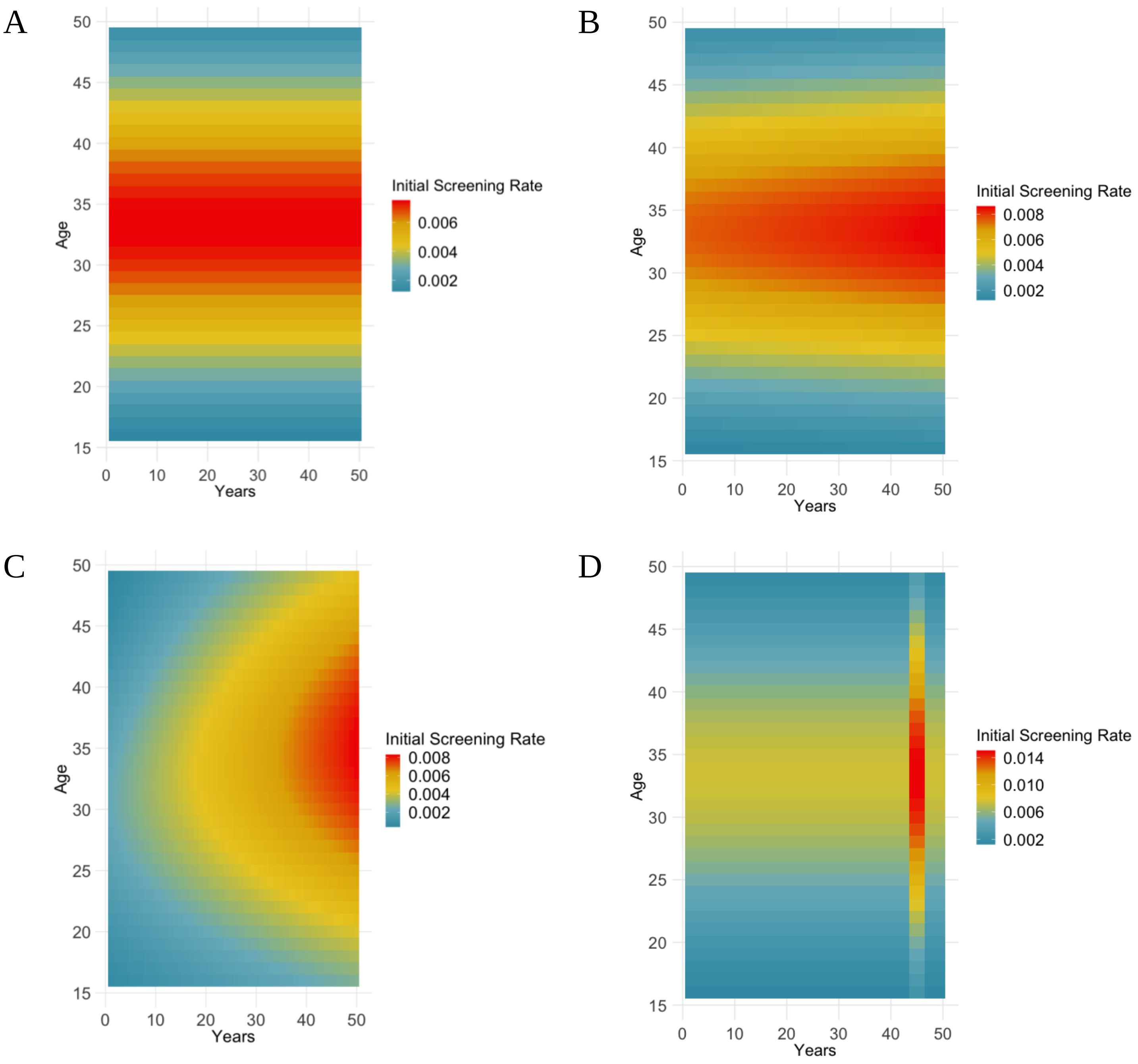

Fig H. Heat map of simulated first-time screening rates across 50 years for women 15-49 years.
Panel A) screening rates that remain constant through time but that increases with age up until age 35 years (i.e., age effects but no cohort effects). Panel B) screening rates that are slowly changing through time but with strong age effect (i.e., age effects and weak cohort effects). Panel C) screening rates that are changing over time and age (i.e., age and strong cohort effects). Panel D) screening rates that are constant over time but except for a 3-year period where screening is 2-fold greater for all age groups (i.e., no cohort effect but strong period effects).

First-time Screening Rate (λ)	Rate Ratio (ϕ)	Lifetime Screening	Screening in the Past Year

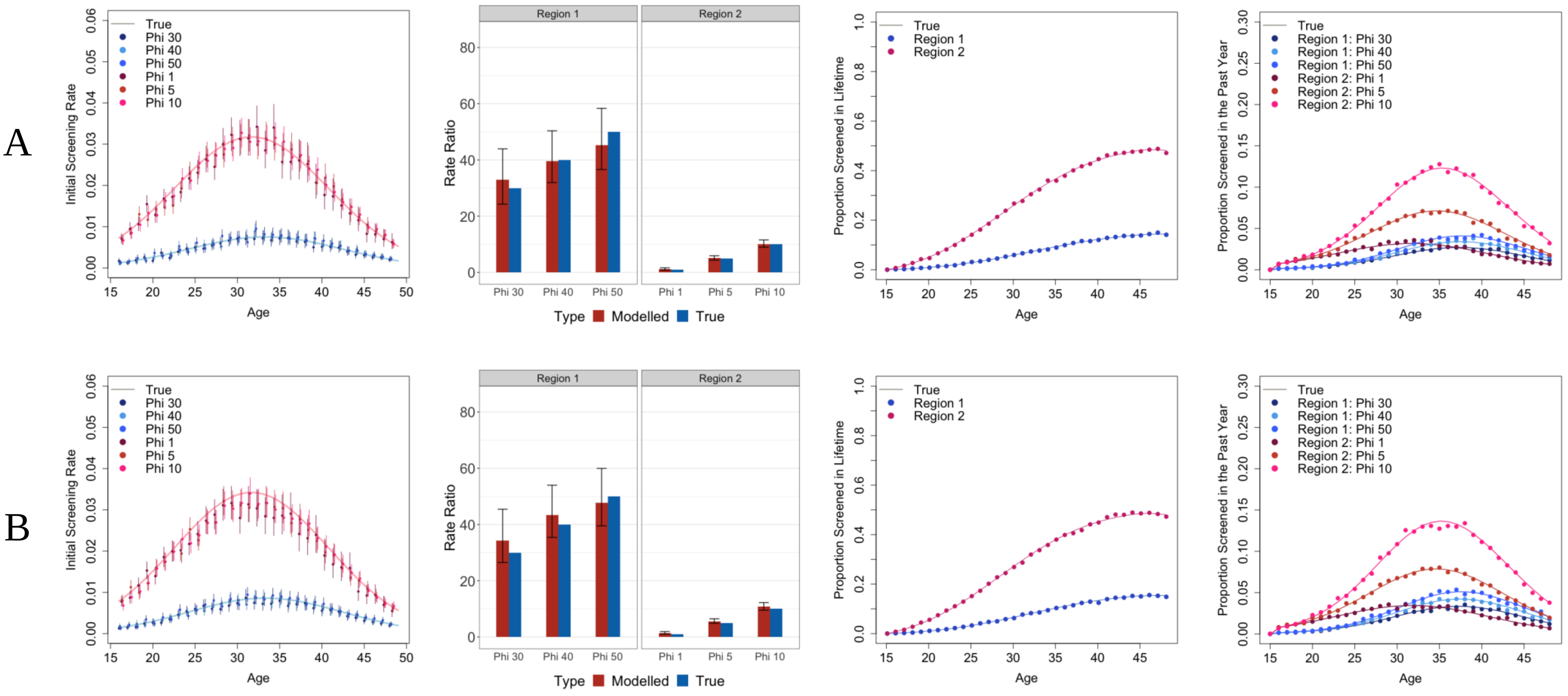

C

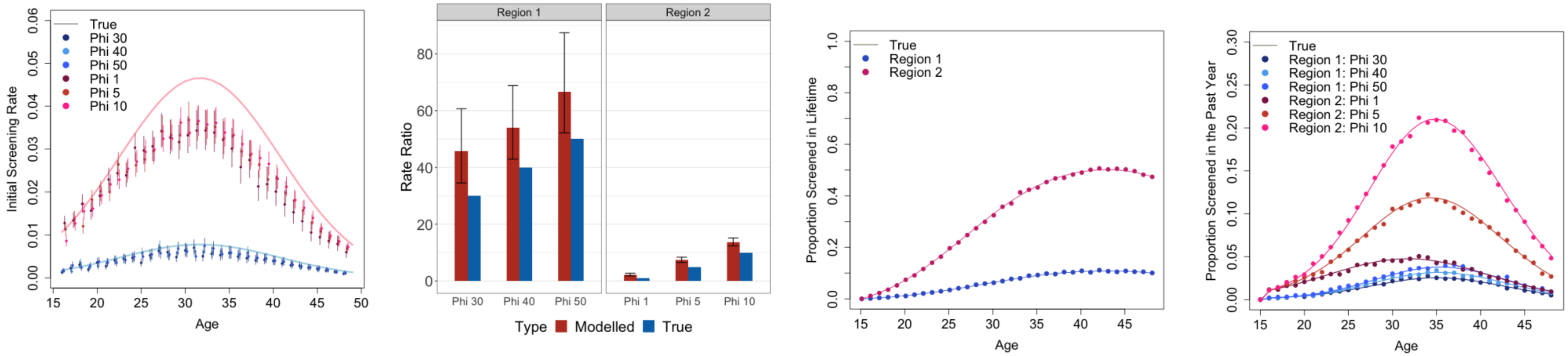
Initial Screening Rate
Age
True
Phi 30
Phi 40
Phi 50
Phi 1
Phi 5
Phi 10
Region 1
Region 2
Rate Ratio
Type
Modelled
True
Proportion Screened in Lifetime
Proportion Screened in the Past Year
Region 1: Phi 30
Region 1: Phi 40
Region 1: Phi 50
Region 2: Phi 1
Region 2: Phi 5
Region 2: Phi 10

D

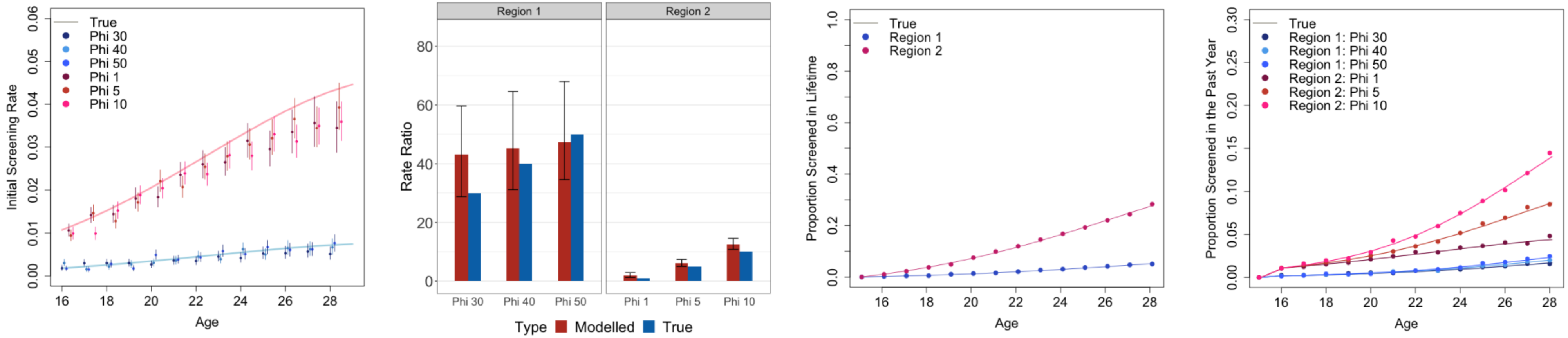
Initial Screening Rate
Age
True
Phi 30
Phi 40
Phi 50
Phi 1
Phi 5
Phi 10
Region 1
Region 2
Rate Ratio
Type
Modelled
True
Proportion Screened in Lifetime
Proportion Screened in the Past Year
Region 1: Phi 30
Region 1: Phi 40
Region 1: Phi 50
Region 2: Phi 1
Region 2: Phi 5
Region 2: Phi 10

E

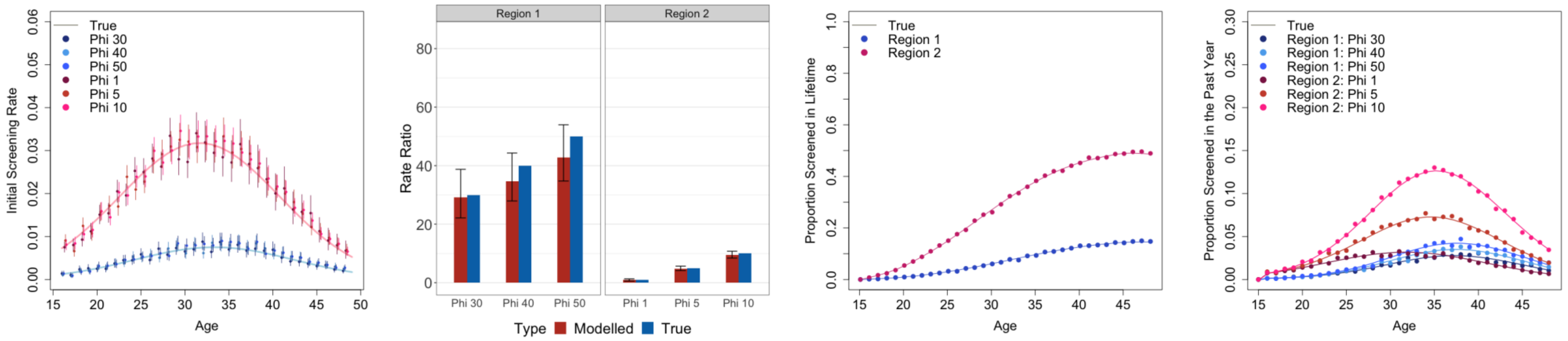

F

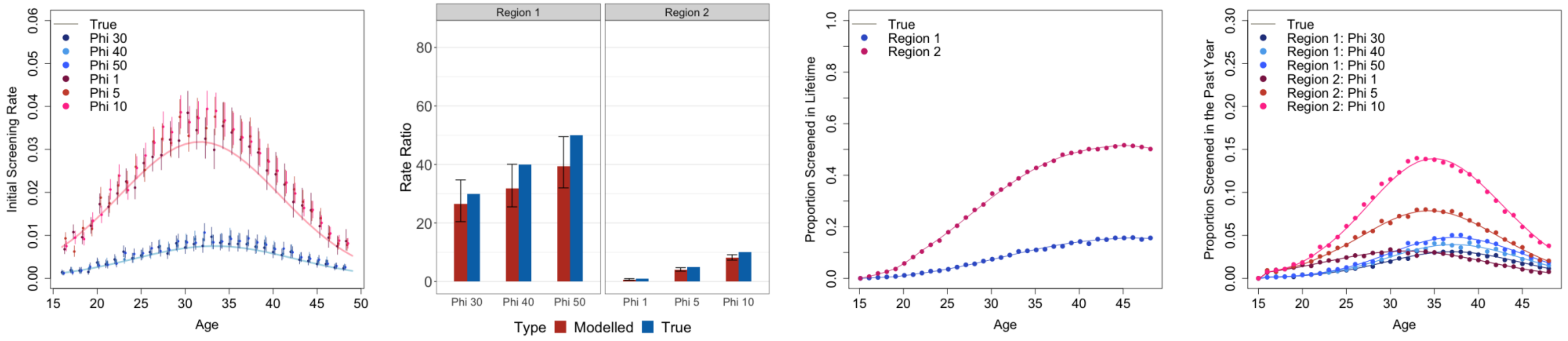

Fig I. Re-screening model simulations comparison of simulated first-time screening rate and rate ratio estimates to modelled estimates.
Six re-screening rate ratios (i.e., 1, 5, 10, 30, 40, 50) belonging to two simulated regions and an overall survey sample size of 10,000 are estimated. Simulations were performed to mimic lifetime and past year screening proportions in Western/Central/Eastern Africa (Region 1) and Southern Africa (Region 2). Panel A) When simulated age-specific first-time screening rates are unchanging for women 15-49 years (i.e., no cohort effect). Panel B) When age-specific first-time screening rates are only minimally changing over time for women 15-49 years (i.e., small cohort effects). Panel C) When there are important changes in age-specific first-time screening rates over time from for women 15-49 years. Panel D) When there are important changes in age-specific first-time screening rates over time for women 15-29 years. Panel E) When age-specific first-time screening rates are unchanging except for a 3-year period where screening rates are increased 1.2-fold for women 15-49 years (i.e., weak period effects). F) When age-specific first-time screening rates are unchanging except for a 3-year period where screening rates are increased 2-fold for women 15-49 year (i.e., strong period effects).

Results for the estimates of re-screening rate ratio

Survey-specific re-screening rate ratios (ϕ_s) were obtained from countries with available survey data (Table E), as well region-specific rate ratios, and an overall rate ratio.

Estimates for the rate ratio parameters were characterized by wide uncertainties. However, all re-screening ratios were large and ranged from 8.3 (95%CrI: 3.9-20.8) in South Africa to 47.1 (18.5, 212.5) in Zimbabwe (Table E). We also report on a sensitivity analysis that adjust for potential telescoping bias. Specifically, we assumed that women would report all screened occurring in the past 18 months as being performed in the last year. Qualitatively, the results for the rate ratio point to high re-screening rates in most countries.

Table E. Estimates of the rate ratio for rate of re-screening for cervical cancer as compared to rate of first-time screening with and without adjustment for telescoping bias.

	**Rate Ratios (95%CrI)**	
**Country**	**Recall period of one year (no telescoping bias)**	**Recall period of 18 months (telescoping bias)**
**Overall**	22.8 (0, 610)	10.7 (0, 476.4)
**Western/Central/Eastern Africa**	34.1 (16.8, 60)	17.4 (6.8, 36)
Benin DHS 2018	32.6 (6, 75)	16.5 (2.1, 47.3)
Cape Verde STEPS 2019	32.4 (5.7, 77.3)	16.5 (2, 50)
Ethiopia PHIA 2018	39.7 (20.2, 96.2)	20.8 (8.3, 60.1)
Ghana SAGE 2007	31.2 (4.7, 69.8)	16 (2, 46.2)
Malawi PHIA 2016	36.5 (18.6, 71.4)	19.1 (7.9, 47.8)
Rwanda PHIA 2019	35.1 (13.5, 83)	17.1 (2.8, 45.1)
Tanzania PHIA 2017	39.0 (19.9, 89.3)	20.5 (8, 57.6)
Zambia PHIA 2016	33.4 (16.3, 68.5)	18 (8.2, 41.5)
**Southern Africa**	21.2 (4.7, 64.9)	8.8 (0.5, 35.4)
Lesotho DHS 2009	17.4 (0.8, 79.8)	6.2 (0, 41.7)
Lesotho DHS 2014	27.1 (5, 136.8)	10.7 (0.1, 100)
South Africa SABSSM 2012	8.3 (3.9, 20.8)	3.9 (1.3, 11.7)
South Africa SAGE 2007	9.6 (0.5, 84.7)	3.6 (0, 31.3)
Zimbabwe DHS 2015	29.2 (11.6, 99.6)	12.2 (2.7, 50.4)
Zimbabwe PHIA 2016	47.1 (18.5, 212.5)	24.8 (6.5, 114)

(DHS = *Demographic and Health Survey*; PHIA = *Population-based HIV Impact Assessment*; SABSMM = *South Africa National HIV Prevalence, Incidence, Behavior and Communication Survey*; SAGE = *Study on Global AGEing and Adult Health*; STEP = *STEPwise Approach to NCD Risk Factor Surveillance*.)

For this analysis, the Western/Central and Eastern Africa regions were combined (hence the same rate ratio)

Robustness check for the life table methods
To investigate the robustness of our approach for the estimation of the proportion of women screened twice, we compared the estimates of lifetime screening by age 45 years in 2020 from the life table to those of lifetime screening among women aged 40-44 years old obtained from the model from objective 1 (see Text A). Because the age groups differ slightly, as well as the underlying modeling assumptions, we do not expect estimates to be exactly the same. However, they should be relatively similar. Overall, our results suggest that the pooled estimates of lifetime screening from both methods are concordant (Fig J). Country-level estimates with countries with two or more surveys are also presented here. Greater discrepancies exist for the country-level estimates, and they are also characterized by wide uncertainties.

Additionally, sensitivity analyses using various rate ratio values was done (Fig K). Although country-level estimates varied greatly with different rate ratio values, pooled estimates for screening twice in a lifetime did not vary greatly. Altogether, these results suggest that estimates of lifetime testing are generally robust for the pooled estimates.

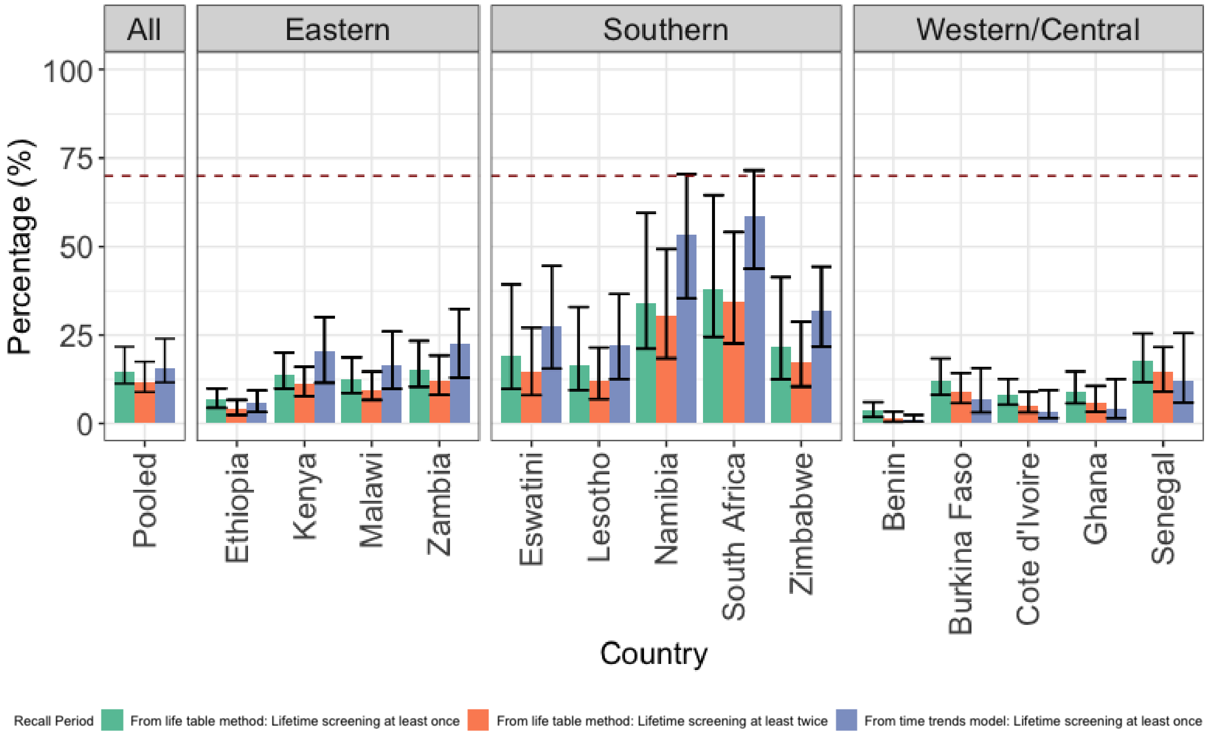

Fig J. Robustness check of estimates for screening twice by the age of 45 years.
This allows for comparison of lifetime screening estimates from the life table method (green bar) to the lifetime screening estimates from the time-trends model (purple bar). Orange bars represent estimates for screening twice. This is done to investigate the robustness of the life table method as the green bars and the purple bars should give comparable estimates.

<table>
<tr><th></th><th>Rate Ratio (ϕ)</th><th>Estimates from Life Table</th></tr>
<tr><td>A</td><td>0.5</td><td>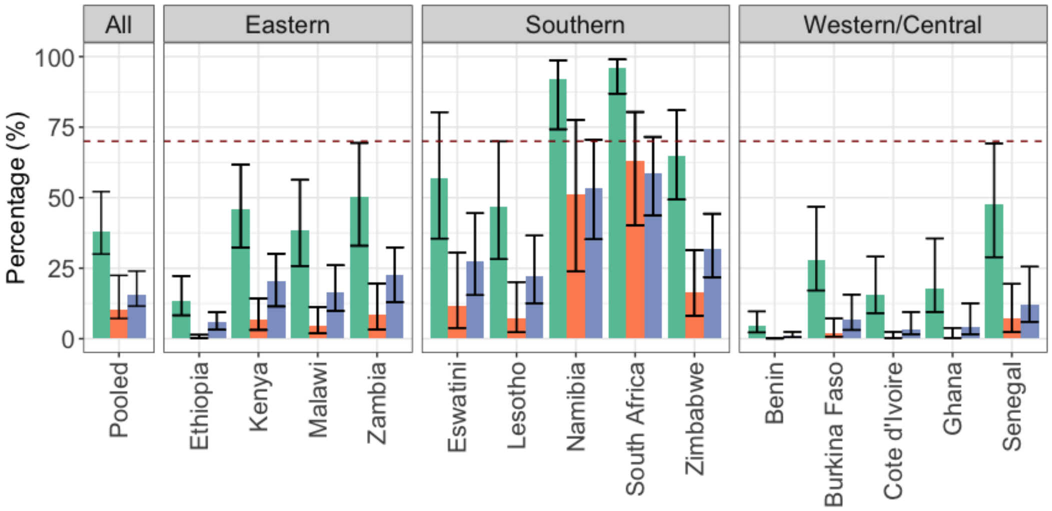
</td></tr>
<tr><td>B</td><td>1</td><td>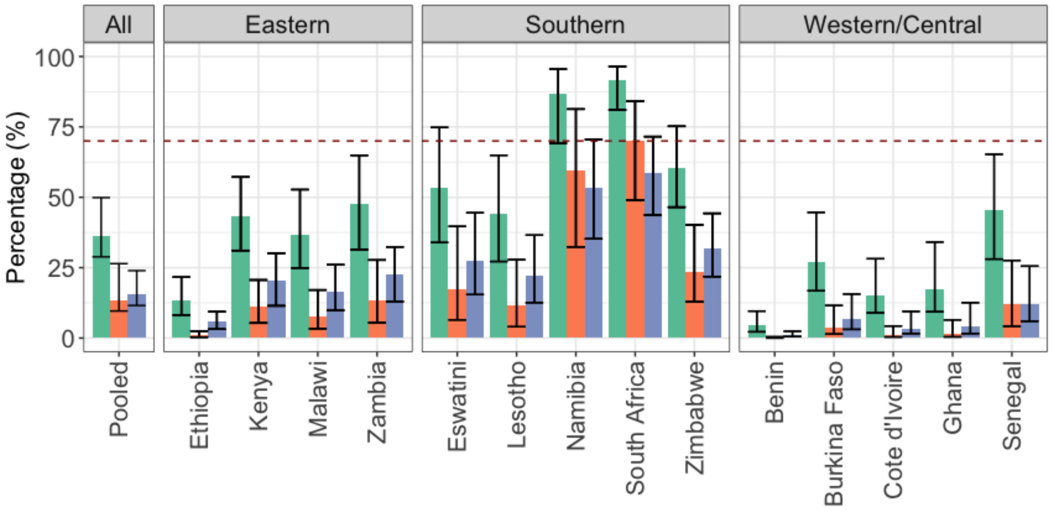
</td></tr>
<tr><td>C</td><td>5</td><td>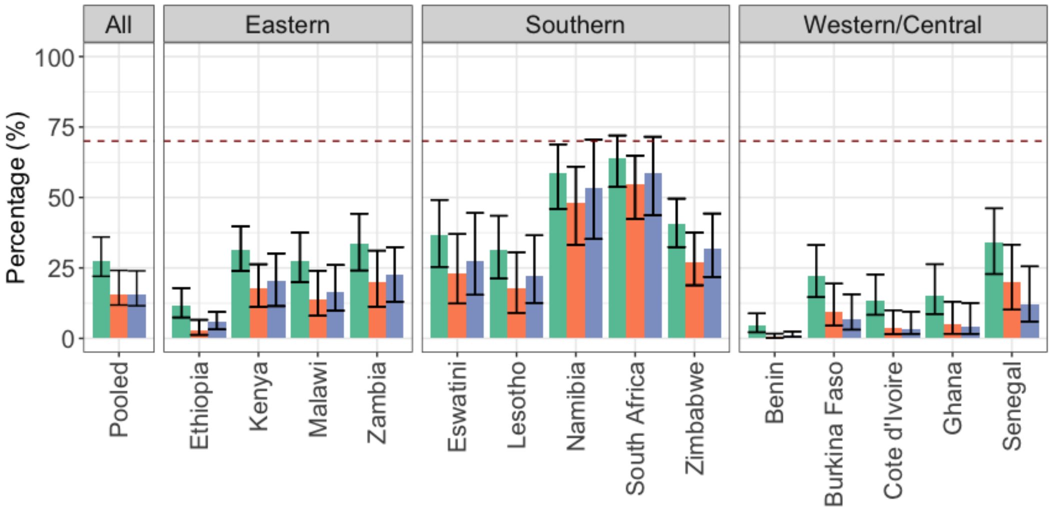
</td></tr>
</table>

D 10

E 30

F 50

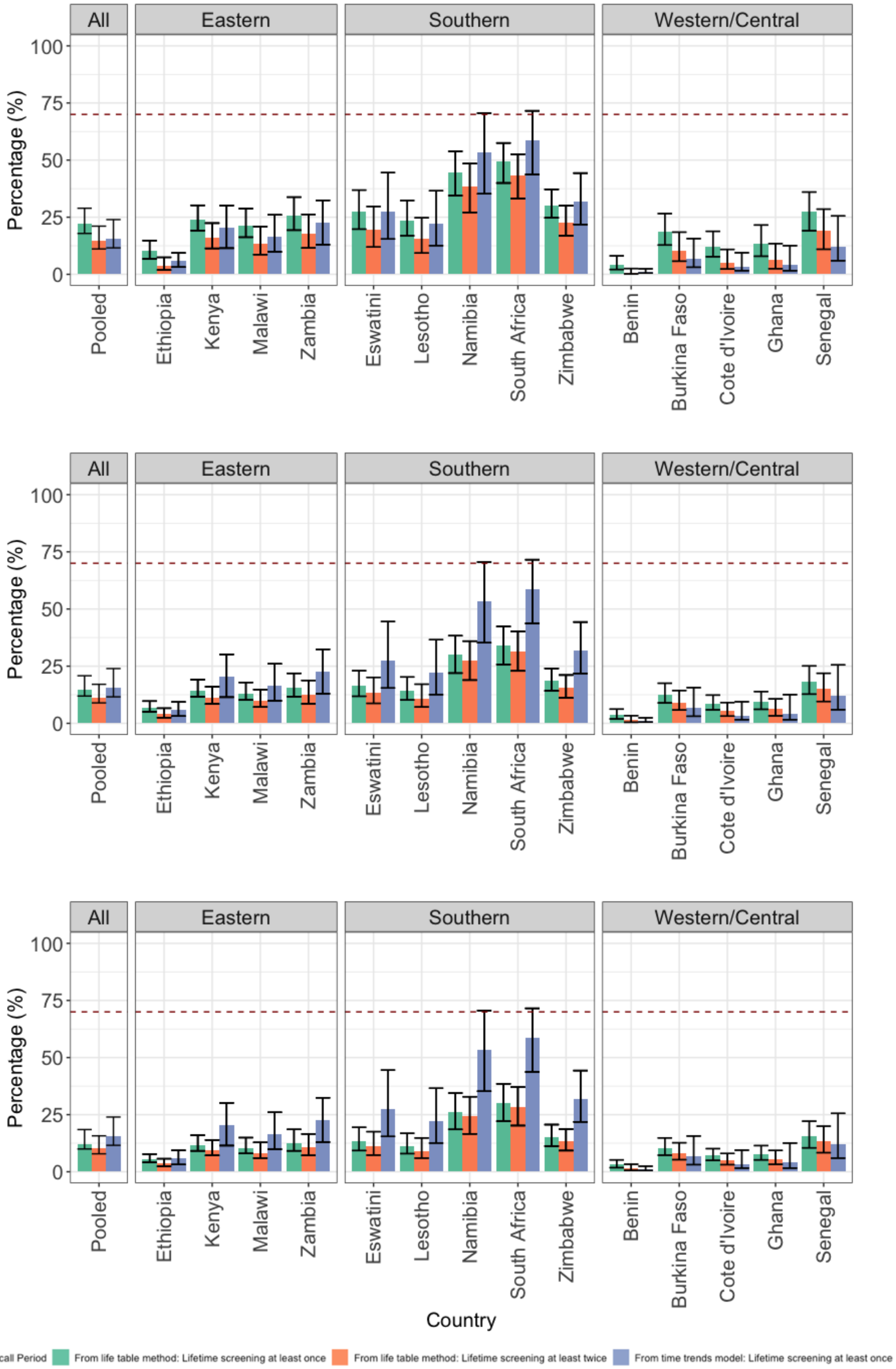

Fig K. Life table methods sensitivity analysis using various rate ratio values.
For panels A to F, one rate ratio value was used to obtain age- and country-specific first-time screening and re-screening rates that were then applied to the life table methods.

Text C. Description of the methods for cervical cancer treatment coverage.

Surveys with information on cervical cancer (CC) pre-cancer treatment coverage is used to estimate the proportion of women treated after receiving a screening result that suggested the presence of a pre-cancerous lesion. These surveys are pooled using a Bayesian meta-analysis approach as described below:

$$Y_i \sim Binomial(N_i,\ p_i)$$

$$logit(p_i) = \alpha + \upsilon_{c[i]}$$

Where Y_i is the outcome for a women's report *i* of pre-cancer treatment coverage and p_i the predicted probability of pre-cancer treatment coverage. This probability is modeled on the logit scale as the sum of an overall intercept (α), and a country-specific random intercept $(\upsilon_{c[i]})$. The model specification is completed using the following priors.

$$\alpha \sim \mathcal{N}(0,\ 10)$$

$$\upsilon_c \sim \mathcal{N}(0,\ \sigma_c) \quad and \quad \sigma_c \sim \mathcal{HC}(0, 3)$$

The overall intercept is given a non-informative prior and the country-level intercept are assumed to follow a normal distribution. The degree of pooling between country is governed by the standard deviation parameters (σ_c) with a half-Cauchy prior.

Table F. Summary of main model assumptions and their justifications.

Objectives	Model Assumptions	Justifications
All	No information or sampling biases.	Despite potential limitations due to the self-reported nature of screening coverage, population-based surveys are representative and have comparable methodology that allow us to track coverage across countries and over time.
	Countries with surveys that ask about a specific screening modality (i.e., Pap smears) have that modality as the primary method of screening.	Data is limited regarding primary screening modalities in many countries partially due to a lack of screening programs in these countries in the early 2000s. Despite this, when data is available the primary screening modalities match those asked in the surveys.
1	Lifetime and past three-year screening coverages are proportional and conditional on age, on the logistic scale through time.	Lifetime screening and screening in the past three years were jointly modelled under this assumption as only 33 surveys had information regarding lifetime screening and 34 on screening in the past three years. Lifetime and past three-year screening were modelled jointly to improve statistical power. Posterior predictive checks suggest good fit to the model.
	Time trends in screening coverage are linear on the logistic scale and vary by regions.	Only half the countries with survey data had two or more surveys to inform trends. Imposing linear trends at the regional level was required because of this data paucity. Here too, posterior predictive checks suggest good fit to the model.
	The odds ratio for the effect of HIV on screening coverage is time and age-invariant.	In several countries, HIV prevalence is relatively low, and we would not have had the required statistical power to model this heterogeneity.
2	The rate ratio between re-screening and first-time screening is time and age-invariant	This assumption was required as our proposed methodology to estimate rate ratio is not able to detect such variations.

References

1. Brooks SP, Gelman A. General methods for monitoring convergence of iterative simulations. Journal of Computational and Graphical Statistics 1998; 7(4): 434-55.
