# Supplementary material for: Regional and country-level trends in cervical cancer screening coverage in sub-Saharan Africa: A systematic analysis of population-based surveys (2000–2020)
Source: PLoS Med. 2023 Jan 12;20(1):e1004143. doi: 10.1371/journal.pmed.1004143 (PMC9882915; doi:10.1371/journal.pmed.1004143)
Supplement: Attachment — Submitted filename: Yang et al. CC trends Response Letter.docx [file pmed.1004143.s002.docx]

Dear Dr. Callam Davidson,

We want to thank the reviewers for their comments. We are pleased to submit the revised version of our manuscript titled “Regional and country-level trends in cervical cancer screening coverage in sub-Saharan Africa: a systematic analysis of population-based surveys (2000-2020)”

Below we have documented the changes we have made to the manuscript. The main changes are described below:

- Clarified the definition of the rate ratios for the re-screening analysis.
- Included additional sensitivity analyses which investigated the role of the 2003 World Health Surveys.
- Adjusted the wording of certain sections, following the reviewers’ suggestions.
- Added relevant items to the discussion and limitations sections of the paper.

We strongly believe that the paper has been improved following incorporation of reviewer and editor suggestions.

Sincerely,

Lily and Mathieu Maheu-Giroux, on behalf of all co-authors

**Reviewer comments:**

**Requests from editors**

| **Comments** | **Response** |
| --- | --- |
| Please include continuous line numbering throughout your manuscript to facilitate review. | This change has been made. We wanted to note however that under certain settings with track changes, the line numbers change |
| Please update your title to ‘Regional and country-level trends in cervical cancer screening coverage in sub-Saharan Africa (2000-2020): a systematic analysis of population-based surveys | This change has been made. |
| In your Data Availability Statement, please confirm whether the data are freely available on request from the sources provided, or whether restrictions are in place. | This information will be added to the Data Availability Statement |
| Please include the study design (a systematic analysis of population-based surveys) In your Abstract Methods and Findings. | This change has been made. |
| At this stage, we ask that you include a short, non-technical Author Summary of your research to make findings accessible to a wide audience that includes both scientists and non-scientists. The Author Summary should immediately follow the Abstract in your revised manuscript. This text is subject to editorial change and should be distinct from the scientific abstract. | An author summary has been added to the revised manuscript, following the Abstract. |
| Thank you for providing the GATHER checklist. Please update Table S6 to use section name/paragraph number rather than page number. | This change has been made. |
| Some of the text in Figure 1 is very small – please enlarge. | This change has been made. |
| Please remove the ‘Role of the funding source’ section. | This change has been made. |
| I could not find details of the specific consent procedures in Table S1. | The consent procedures were the same for all surveys. We referred to table S1 for the list of all surveys, including their report’s reference where the details of the consent procedures are provided. Further, we added this footnote to Table S1.  *“Informed consent was obtained from all participants or their guardians for all surveys, following verbal communication from the surveyors describing the purpose and scope of surveys. To protect the privacy and safety of participants, data is then anonymized, and researchers must request and be approved for use of data before access is granted to surveys. More specific details regarding consent procedures may be found in specific country reports.”* |
| Please define the meaning of the shaded area in the Figure 3 legend. | The shaded areas represent the 95% credible intervals. This has been added to the Figure’s description. |
| Please confirm that the appropriate usage rights apply to the use of the map in Figure 4. Please see our guidelines for map images: | The base layer for the map was obtained from the “world” dataset from the spData R package which takes their data from Natural Earth. The Natural Earth terms of use states that no permissions or acknowledgements are required to use it. The appropriate information was added to the bottom of the figure:  *“The base layer for this figure was obtained from the “world” dataset from the spData R package which takes their data from Natural Earth.”* |
| Please define the meaning of the error bars in the Figure 5 and Figure 6 legends. | The error bars represent the 95% credible intervals. It has been added to the Figure description. |
| Please remove the Data Sharing, Declaration of interests, and Funding sections and instead ensure that all relevant information is captured in your responses to the respective sections of the submission form. | This change has been made. |
| Please use ‘et al.’ only after listing the first six authors in your references. | This change has been made. |
| Please include ‘[preprint]’ in reference 15. | This reference is now published, and the reference has been updated appropriately  *“Maheu-Giroux M, Sardinha L, Stöckl H, Meyer SR, Godin A, Alexander M, et al. A framework to model global, regional, and national estimates of intimate partner violence. BMC medical research methodology. 2022;22(1):1-17.”* |

**Reviewer #1**

| **Comments** | **Response** |
| --- | --- |
| Abstract | |
| Background: term "... monitored re-screening by age 45" is not clear. % screened once and % screened (at least) twice looks easier to understand.  "High HIV prevalence setting" is not defined in the abstract. | These changes have been made and “High HIV prevalence setting” was removed from the abstract. |
| Introduction | |
| Together with ref 2 (dedicated to all cancers), the authors could cite the paper, dedicated to cervical cancer only: <https://www.ncbi.nlm.nih.gov/pubmed/31812369>  First sentence of 2nd §.  A more international ref could be given to illustrate the trends of CC and how it changed through screening for instance (<https://www.ncbi.nlm.nih.gov/pubmed/19695864>  ).  One of the  refs that illustrates the impact of HPV vaccination on incidence of CC in young women: <https://www.ncbi.nlm.nih.gov/pubmed/34741816> | Thank you for the suggestion, these references have been included in the introduction. |
| Last sentence of §3. Despite the importance of screening frequency in the WHO recommendations, little data exists on re-screening frequency in sub-Saharan Africa. Also on the % screened once there is little data. Suggestion:" little data exists on the proportion of women who were screened once or two times." | This suggestion was incorporated. |
| The introduction does not clarify "High HIV prevalence setting".  This should be explained in the Introduction, or maybe the title should be changed.  By reading the title I had the impression that settings (HIV clinics, sexual health centres, …) were the target of the paper.  After reading the Intro, I understood that the whole female population of sub-Saharan African countries (where HIV prevalence is high) is the target. | “High HIV prevalence setting” was removed from the title |
| Material & Methods | |
| Data processing Page 4, line 2: treatment outcome: this usually means the % where treatment was successful or failed.  Better wording would be: percentage treated or treatment coverage. | This suggestion has been incorporated. |
| Regional and national estimates of cervical cancer screening coverage time trends Did the authors did not have one dbase where observed 2nd screening data were available to validate the modeled estimates? | No, unfortunately none of the available data provided information on observed or self-reported 2^nd^ screening, despite the importance of this metric. |
| Given the large number of figures, I suggest to move Fig 2 to the supplementary materials. The colors used could be more contrasting to increase read-friendliness. | We have 6 figures in the manuscript. We believe that Figure 2 provides useful insights for readers, as it easily present the number, timing, and types of surveys available by region. If the editor agrees with this reviewer, we will move it with to the appendix.  We have changed the colours to be more contrasting however |
| Authors present pooled estimates for sub-continents and for the whole SSA.  Was a weighting used (proportional to the population size of the countries providing data) or was the pooling done using study size weights.  A population weighting could be considered if not done. | Yes, weighting was used to obtain aggregated estimates (e.g., age, country, region). Using post-stratification, we used the population denominators as weights. Wording of the manuscript was slightly adjusted to make this clearer and avoid further confusion.  *“In other words, when pooling strata (i.e., country, year, five-year age group, and HIV serostatus), the population weight of each stratum was considered.”* |
| The authors should provide definition of "rate ratios" clarifying numerator and denominator, used to compute the proportion women ever screened who had at least 2 screenings. | Clarification of what the definition of the rate ratio is has been added to the methods  *“Our preliminary analyses suggested that the rate of first-time screening and the rate of re-screening were not the same. As such, we first estimated a rate ratio between the rate of re-screening (i.e., the rate at which women already screened once are screened again) and the rate of first-time screening for each region.”* |
| Results | |
| Figure 3. Please add information on the credibility intervals.  Figure 4: the contrast between color schemes is weak.  Please use a stronger contrast scheme. | The information on the credibility intervals was added. Thanks for pointing this omission.  We changed the color of Figure 4 to increase contrast. |
| Coverage of cervical cancer screening by HIV serostatus Page 14, last line of §1: "ORs were close to the null".  Should be changed to ".. close to unity". | We have replaced “null” with “unity”, as suggested. |
| Proportion of women aged 45 years of age screened at least twice Given the lack of definition of "rate ratio" in the Methods it is difficult to understand "…we estimated that the rate ratio of re-screening versus first-time screening was largely above one in all instances.".  I intuitively expected a value <1, meaning that not all ever screened women would have had a 2nd one.  However, after reading the Results it becomes that my first intuitive understanding was wrong. | Alongside clarifying the definition in the methods’ section, we also added a line in the results to clarify what this means  *“That is, women who have already been screened once are screened again at a greater rate than women who have never been screened.”* |
| Title of Figure 5. "Estimates of women aged 45 years in 2020…". Change into: "Estimates of the percentage of women aged 45 years in 2020… among all women in the target age range".  Credibility intervals are shown but not explained in the figure title. | We have followed the reviewer’s suggestions. |
| Discussion | |
| Page 17, §2 "vaccinations are not prophylactic", change into vaccinations are only prophylactic. | This change has been made. Thanks for noticing this typo. |
| I am missing the following three discussion points: - probably the screening coverage and treatment coverage estimates are better in countries with data compared to those without data; so the pooled estimates might be worse for the whole SSA -HPV testing on self-samples offers opportunities to increase coverage as concluded from a recent meta-analysis (<https://www.ncbi.nlm.nih.gov/pubmed/30518635>) including two trials conducted in Africa demonstrating its effectiveness. - surveys collecting information regarding self-reported screening experience generally overestimate over-estimate screening coverage. | Thank you for these suggestions, we agree with the reviewer that these are important points and have included them in the discussion section. |

**Reviewer #2**

| **Comments** | **Response** |
| --- | --- |
| The abstract states that an estimate 14% of women aged 30-49 had ever been screened in 2020. The credible interval is given as 11-22%. However, in the results, on page 11 and in Table 1, the CI is given as 11-21%. I suggest a thorough QC. | Yes, this was indeed a typo. |
| The abstract describes rescreening rates as high, and then states that an estimated 12% of women had been screened twice by age 45 in 2020. Whilst I can see what is meant, this is confusing at first, since 12% is not "high". | The phrasing in the abstract has been changed to avoid further confusion. |
| Looking at Figure 2, it is notable that almost all the surveys prior to 2010 were from the WHS in 2003, with only four other surveys in that decade. Could this concentration of information around 2003 have an undue influence on the estimated time trends in the data? Were any sensitivity analyses carried out, perhaps looking only at data from 2010-2020? Perhaps a series of sensitivity analyses could be done, excluding each survey type in turn? | It is a limitation of the analysis that most of the surveys available were carried out after 2010. Due to the limited amount of available data however it is difficult to conduct analyses which exclude each survey type. To address the reviewer’s point, we conducted two additional analyses. For the first one, we only include surveys from 2010-2020 to see if the temporal trends are different. Secondly, we considered all data but adjusted for the WHS survey using a dummy variable. For both analyses, we found that our conclusions are robust, and the results are qualitatively similar. Results are presented in Figure S6.  *“Additionally, a sensitivity analysis was done to investigate the impact of utilizing only surveys conducted after 2010. Overall, the general qualitative trends and low screening coverages remain the same. However, there is a slight change in the direction of the trend for Western/Central Africa, reflecting the lack of repeated surveys in that region. In a second sensitivity analysis, we included fixed-effect in the model to adjust for the World Health Survey –the most common survey conducted in the early 2000s. Results of this analysis saw minimal change from our main analysis.”* |
| The data sharing statement includes a link to a clean dataset and program, but the link did not work for me. This is not critical to my review - I was looking out of interest - but it will need to be fixed. | The link to the data sharing has been fixed, thanks for letting us know. |

**Reviewer #3**

| **Comments** | **Response** |
| --- | --- |
| General | |
| The authors often refer to "treatment" or "CC treatment" instead of "cervical pre-cancer treatment." To avoid confusion, the authors should make sure to refer to pre-cancer treatment throughout all sections of the manuscript including the supplemental material. | We have followed the reviewer’s suggestion and this change was implemented throughout the manuscript. In some instances, we left it as “treatment” when the sentence discussed the importance of treatment in general. |
| It would be helpful if all supplemental materials were referenced in the manuscript. | Yes, all supplemental materials are now referenced. |
| Abstract | |
| Under "Methods and Findings" it would be helpful if the authors made clear that these were cross-sectional and not longitudinal surveys. | This change has been made. |
| It would be good to mention the much higher screening coverage in Southern Africa compared to the other African regions. | We now mention the higher screening coverage in Southern Africa. |
| The sentence "Rescreening rates were high, and it was estimated that 12% (95%CrI: 10-18%) of women had been screened twice or more by age 45 in 2020" is a bit confusing, as 12% does not seem high. I suggest rephrasing to clarify that the high rescreening rates refer to previously screened women, whereas the 12% refer to all women. | This change has been made. |
| Introduction | |
| The word "between" in the last sentence of the introduction section can be deleted. | The word was deleted. |
| Methods | |
| Data on "screening in the past year" extracted from the surveys but not really used in this analysis? Or was this information used in the life table method? | Yes, correct. The data for screening in the past year was used for the life table method. |
| "Treatment outcomes" is not an ideal term for "treatment after positive test result for pre-cancer" as many readers will think of outcomes after pre-cancer treatment such as disease recurrence, adverse events, etc. | Phrasing of this term has been changed throughout the paper. |
| The limited data on screening modalities and cervical pre-cancer treatment approaches should also be acknowledged in the limitations section of the discussion (not only in the methods). | We agree. This has been added to the limitations section.  ***“****Similarly, the limited data regarding screening modalities and treatment approaches prevent their more detailed analysis”* |
| The authors state that country-level estimates were only presented for countries with at least two surveys. Did the two surveys have to assess the same CC screening recall period (lifetime vs past 3 years) or not? | No, the survey did not need to have the same recall period as we felt that our adjustment factors, which varied by age groups, were suitable to correct for such differences. |
| The sentence "Our preliminary analyses suggested that first-time screening and re-screening were independent" needs further explanations. It seems counterintuitive that first-time screening and re-screening are independent. | Thanks for pointing out this incoherence. We have changed the sentence for clarity. |
| Pre-cancer treatment information was also self-reported, correct? | Yes. This was clarified in the methods  *“This data was all self-reported and the specific questions that were asked can be found in Table S1 of the supplemental materials.”* |
| Results | |
| Would it be possible to show country-level CC screening trends stratified by both HIV status and recall period (lifetime vs past 3 years)? Typically, shorter screening intervals are recommended for women living with HIV. Therefore, it would be interesting to see how HIV status impacts CC coverage estimates by recall period. Or if not the trends, then maybe the CC coverage estimates reported in Table 1 could be stratified by both recall period and HIV status? | We have added to Table 2 the estimates we’ve obtained for screening in the past 3 years among WLHIV. |
| The authors found that in Southern Africa the odds of screening were similar among women with and without HIV. Does this only refer to lifetime screening or also screening in the past 3 years? | This refers to both screening ever and in the past 3 years. We used a multiplicative model (logit scale) such that the effect of HIV on CC screening is proportionally the same for lifetime and past three years screening. |
| If the OR in Table 2 refers to "ever screened" (i.e., lifetime screening?), does it make sense to adjust for recall periods? | Our model adjusts for recall periods so that we can pool all the available data. In Table 2, we mention that we adjusted for recall period since some of the observations pertain to that recall period. However, we present results for “ever screened”. |
| Would it be possible to stratify the estimates in Figure 5 by HIV status? As more frequent screening is recommended for women living with HIV, it would be interesting to see how HIV status impacts the re-screening rates. | This is something we initially sought to do. Unfortunately, after stratifying the estimates by HIV status, the resulting sample sizes per one-year age groups are simply too small to conduct our analyses (i.e., the uncertainty blows up). |
| It would be interesting to know a bit more about how the pre-cancer treatment coverage was assessed in the 4 available surveys. Did they ask about pre-cancer treatment within a certain time frame or at any point after a positive CC screening result? | Questions asked include whether treatment was received in general or whether treatment was received on same day or different day. A sentence was added in the methods to direct readers to the Table S1 where this information can be found.  *“This data was all self-reported and the specific questions that were asked can be found in Table S1 of the supplemental materials.”* |
| The sentence "As this analysis was limited to women who had been screened and had received their screening result, the true number of women with pre-cancerous lesions who are treated is likely to be lower" rather belongs into the discussion section. | This sentence has been removed from the results section. |
| Discussion | |
| "Despite the importance of primary prevention, vaccinations are not prophylactic and will not cure women already infected with HPV." I assume the authors mean "not therapeutic" rather than "not prophylactic" (or want to delete the "not"). | Yes, the reviewer is correct. We have corrected this. |
| The authors mention that service integration could partially explain why they found higher CC screening coverage among women living with HIV in certain regions. The authors may also want to mention that earlier and more frequent screening is recommended for women living with HIV which may affect coverage estimates particularly among the younger age group. | Thank you for the suggestion, this point was added to the discussion.  ***“****With the increased recognition of the additional risks of CC development in WLHIV, recognition of WLHIV as a priority group for CC prevention, HIV/CC service integration has been implemented in many countries in Africa.”* |
| Could the authors elaborate more on potential reasons for the higher CC screening coverage in Southern Africa compared to the other regions? | Yes, we added such a sentence in the discussion.  *“Greater financial resources, more developed healthcare infrastructure, and historical attention to cervical cancer screening priorities may have contributed to higher screening coverage in Southern African countries.”* |
| The analysis is based on self-reported information. How do the authors expect self-reporting to affect their estimates? Should this be included in the limitations section? | A sentence regarding the potential effect of self-reporting and overestimation was added to the limitations section.  *“Finally, our analysis relied on self-reported measures of screening and treatment coverage. Due to biases such as recall and social desirability bias, this may potentially lead to overestimation of our estimates”* |
| The authors should highlight in the limitations section that their assessment of pre-cancer treatment coverage is based on 113 women only. | This has been highlighted in the limitations section.  *“Furthermore, the treatment analysis was based on the self-reports of only 113 women.”* |
| Data sharing | |
| It is great that the authors make the code available on GitHub. Unfortunately, the provided link to the GitHub repository does not work. Is it really publicly accessible? | The link to the repository has been fixed and appropriately added to the data availability section |
