# Supplementary material for: Regional and country-level trends in cervical cancer screening coverage in sub-Saharan Africa: A systematic analysis of population-based surveys (2000–2020)
Source: PLoS Med. 2023 Jan 12;20(1):e1004143. doi: 10.1371/journal.pmed.1004143 (PMC9882915; doi:10.1371/journal.pmed.1004143)
Supplement: Attachment — Submitted filename: Yang et al. CC response to reviewers.docx [file pmed.1004143.s003.docx]

Dear Dr. Callam Davidson,

We want to thank you for your email and the reviewers again for their comments. We have addressed all queries in the updated revised manuscript.

Sincerely,

Lily and Mathieu Maheu-Giroux, on behalf of all co-authors
